# Cloaking of solar cell contacts at the onset of Rayleigh scattering

**DOI:** 10.1038/srep28669

**Published:** 2016-06-24

**Authors:** Etor San Román, Alan Vitrey, Jerónimo Buencuerpo, Iván Prieto, José M. Llorens, Antonio García-Martín, Benito Alén, Anabil Chaudhuri, Alexander Neumann, S. R. J. Brueck, José M. Ripalda

**Affiliations:** 1 IMM-Instituto de Microelectrónica de Madrid (CNM-CSIC), Isaac Newton 8, PTM, E-28760 Tres Cantos, Madrid, Spain; 2 Center for High Technology Materials, University of New Mexico, Albuquerque, NM 87106, USA

## Abstract

Electrical contacts on the top surface of solar cells and light emitting diodes cause shadow losses. The phenomenon of extraordinary optical transmission through arrays of subwavelength holes suggests the possibility of engineering such contacts to reduce the shadow using plasmonics, but resonance effects occur only at specific wavelengths. Here we describe instead a broadband effect of enhanced light transmission through arrays of subwavelength metallic wires, due to the fact that, in the absence of resonances, metal wires asymptotically tend to invisibility in the small size limit regardless of the fraction of the device area taken up by the contacts. The effect occurs for wires more than an order of magnitude thicker than the transparency limit for metal thin films. Finite difference in time domain calculations predict that it is possible to have high cloaking efficiencies in a broadband wavelength range, and we experimentally demonstrate contact shadow losses less than half of the geometric shadow.

The contacts in optoelectronic devices have two competing requirements: low electrical resistivity, and high light transmittance. To date, this trade-off has limited the performance of optoelectronic devices such as solar cells and light emitting diodes (LEDs). The first proposals to direct light away from the top contacts appeared not long after the birth of photovoltaics, but these schemes based on ray tracing optics (such as microlenses) suffer from added complexity, and fail to work when there are features not much larger than the light wavelength^1^,^2^. Recent advances in nanowire technology have improved the contact characteristics of low power density devices^3^,^4^, but still result in shadow losses that are unacceptable for concentrator solar cells and other high power density devices such as LEDs. For devices operating at specific wavelengths (which is not the case for solar cells), this difficulty can perhaps be overcome by engineering plasmonic resonant effects^5^,^6^. Transparent conductive oxides such as indium tin oxide (ITO) offer sufficient performance (90% transmission with an 11 Ω/sq sheet resistance) for devices operating at low current densities, such as flat panel displays and organic LEDs^7^. The scarcity of Indium has motivated research into alternatives such as the organic polymer PEDOT:PSS, with 91% transmission and 226 Ω/sq sheet resistance^8^. Van de Groep *et al*. have demonstrated silver nanowire network arrays on glass with 89% transmission and 20 Ω/sq sheet resistance^9^, and the group of Prof. Cui has perfected metal nanowire fabrication from electrospun fibres, reaching 90% transmittance and 2 Ω/sq sheet resistance^4^. State of the art multijunction solar cells require noble metal top contact grids with >95% transmittance and <10 Ω/sq sheet resistance to operate at high sunlight concentrations (>500 suns)^10^,^11^. Here we demonstrate nanowire arrays with a sheet resistance of 2.5 Ω/sq at 96.4% transmission (with a long period array), and 0.31 Ω/sq at 91% transmission (with a short period array). We show that optimized metal nanowires cast an effective shadow much smaller than the geometric shadow, and consequently, optoelectronic devices can be designed in a way that significantly reduces the losses due to the top contact, overcoming the previously mentioned trade-off. The effect is applicable to a wide range of technical problems, such as contacts in solar cells, light emitting diodes (LEDs) and flat panel displays, but here we focus our attention on concentrator photovoltaics (CPV), perhaps the most demanding of these due to the need to minimize losses across a broadband wavelength range.

Recent interest in plasmonic solar cells has primarily been focused on increasing the optical path length or near field intensity in optically thin solar cells by adding components such as nanoparticles or diffraction gratings^12^,^13^,^14^. Instead, we focus on nanostructuring an already existing and necessary component, the front contact, to reduce shadow losses^12^.

The questions addressed in this work are best understood on the basis of the analytical solution for the interaction of the electromagnetic field with a freestanding metal cylinder^15^,^16^. In the small size limit (when the ratio of the wire circumference to the wavelength is *x *≪ 1), under normal incidence, and away from resonance, the scattering amplitudes for light polarized parallel to the wire *T*_*p*_ and transversal to the wire *T*_*t*_ can be approximated to^15^:




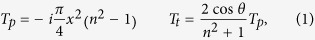




where *θ* is the scattering angle, *n = n*_*w*_/*n*_*i*_ is the relative refractive index, *n*_*w*_ is the refractive index of the wire, *n*_*i*_ is the refractive index of the incidence medium, *x = πwn*_*i*_*/λ* is the size parameter, *w* is the wire diameter, and *λ* is the free space wavelength. Of interest here are the absorption and scattering efficiencies defined as the absorption and scattering cross sections divided by the wire area projected onto a plane perpendicular to the incident beam (geometric shadow). The optical theorem states that the total extinction (absorption+scattering) efficiency is directly given by the forward scattering amplitude as 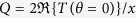
17. The scattering efficiencies in the small size limit are^15^:




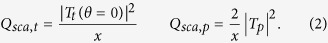




Therefore *Q*_*sca*_ scales with size as *x*^*3*^ while *Q* scales as *x*, implying that the extinction is dominated by absorption in the small size limit. As *x* → 0, both the extinction efficiency and the wire geometric area tend to zero as *x*, while the extinction cross section tends to zero as *x*^*2*^. Therefore in the small size limit, and in the absence of resonances, metal nanowires become invisible, a fact that can be used to advantage in the design of top contact grids for solar cells and LEDs. At resonant wavelengths the Rayleigh approximation is of little use, as the series expansion of the scattering amplitudes in terms of powers of *x* cannot be truncated at the leading terms, except for extremely small values of *x*, and the resulting trend as a function of *x* is completely different from that given by Rayleigh. Neglecting dissipative losses, the extinction efficiency due to a localized surface plasmon resonance in a free standing cylinder under normal incidence illumination is always *Q* = 4*/x* at the resonant wavelegth.^16^ This is in clear contrast with Rayleigh scattering, for which case *Q* is proportional to *x*. The opposing trends of resonant and non-resonant contributions to extinction, will be discussed again below, in the interpretation of our results.

To study light scattering and absorption in geometries relevant to state of the art solar cells, we have used finite difference time domain calculations (FDTD). We have calculated the effective shadow factor as *S* = (*J*_*0*_−*J*)/*J*_*0*_, where *J* and *J*_*0*_ are the fluxes entering the active part of the semiconductor with and without the metal nanowires, respectively. The shadow efficiency is *S*/*G* where *G* is the fraction of the device area covered by the contacts (geometric shadow factor) and the cloaking efficiency is given by 1−*S*/*G*. The shadow efficiency as a function of wire width for a realistic device geometry (Fig. 1a) and unpolarized light is shown in Fig. 1b. The horizontal stripes are diffraction effects with little impact in the total generated photocurrent. Localized surface plasmon resonances can be recognized in Fig. 1b as oblique bands with increased shadow losses at nearly constant size parameter (*x = πwn*_*i*_*/λ*). These are most easily recognizable at wavelengths from 800 to 1000 nm. For wire widths around 400 nm, the shadow is well below the geometric limit, with cloaking efficiencies above 50% for most wavelengths.

Underlying the complex patterns in Fig. 1b due to the combined effects of diffraction, non-resonant extinction, and localized surface plasmon resonances, there is a simple trend that is revealed by integrating across wavelengths. We have weighted the data in Fig. 1b with the direct solar spectrum^18^ to find the width that should yield the highest sunlight transmission. The resulting spectrally weighted shadow efficiencies as a function of wire width are shown in Fig. 1c. The fact that the shape of the weighted shadow efficiency curve can be fitted to *ax + b/x* using only two adjustable parameters is not coincidental, and still holds for systems with different composition and geometry (see supplementary information). The Purcell sum rule, closely related to the Thomas-Reiche-Kuhn sum rule, determines that if the integration interval is extended to infinite wavelength, the resulting spectrally integrated extinction efficiency is given by a single term directly proportional to the wire diameter^15^,^19^. In the present case of a finite integration range weighted by the solar spectrum, the minimum in the integrated shadow efficiency curve of Fig. 1c is due to the crossover between resonant extinction (proportional to 1/*x*) predominating at short wavelengths, and non-resonant Rayleigh scattering (proportional to *x*) predominating at long wavelengths. The integrated shadow efficiency deviates from the Rayleigh approximation for wires wider than 500 nm, and asymptotically tends to the ray-tracing limit.

To experimentally demonstrate the described cloaking effect, we have fabricated GaAs solar cells with near optimal contact wire widths. The fabricated devices and the corresponding experimental results and simulations are shown in Fig. 2. At long wavelengths, cloaking efficiencies higher than 60% have been obtained for silver nanowire arrays (Fig. 2b). The high absorption at short wavelengths that gives gold its characteristic yellow colour causes much higher losses than in the case of silver (compare Fig. 2b,e), but the cloaking effect still occurs at long wavelengths. The difference in period has little effect on the cloaking efficiency, but is the result of two different fabrication processes that were developed in parallel (see Methods and Suppl. Info.). The main differences between our simulations and experiments occur in the case of silver wires at long wavelengths for parallel polarization (Fig. 2c,f). Reflection is the only relevant optical loss mechanism for a perfect ideal nanowire illuminated with parallel polarised light. (see Figs S1 and S23 in the supplementary material and ref. 16). The main difference between the simulated idealisation and our experimental nanowires is surface roughness. Accurately simulating the effects of surface roughness on the optical properties of metal nanowires would have a very high computational cost, but the effects of roughness can be inferred by comparing the experimental results from rough nano-structures with results from perfect nano-structures without roughness. The optical properties of metal nanowires with various kinds of imperfections such as surface roughness and polycrystalline grain boundaries have been reported by various independent groups to depart from the results expected from ideal, perfect nanowires^20^,^21^,^22^. Most importantly, surface roughness results in the coupling of localised and propagating surface plasmons with radiation polarised along the wire axis, an effect that does not exist for ideal wires lacking structure along the longitudinal direction. Light incident on the wire that would otherwise be reflected is instead coupled to the wire due to surface roughness. It has been shown that most of the parallel polarized light coupled to nanowires on high index substrates such as GaAs is re-emitted towards the substrate, increasing transmission^23^. Billaudeau *et al*. have also shown that periodic perturbations along the propagation path of surface plasmons result in increased transmission to the GaAs substrate^24^. Furthermore, these authors have shown that the minima in the propagation length of surface plasmons coincide with the transmission maxima due to radiative re-emission into the GaAs substrate. This indicates that most of the parallel polarized light that couples to propagating surface plasmons is re-emitted into the GaAs substrate, rather than absorbed by the metal or re-emitted to free space. Therefore, for parallel polarized light, the experimental shadowing efficiencies of real nanowires with surface roughness can be expected to be lower than that of ideal nanowires due to decreased reflection and increased scattering into the substrate of light coupled to surface plasmons. The effect of roughness is more pronounced in our silver wires than in our gold wires because the top side of the later was flattened by an argon ion milling step that was not used in our silver wires.

The silver wire arrays with a period of 4 μm presented in Fig. 2 have a wavelength integrated transmission of 91%, and a measured grid sheet resistance of 0.31 Ω/sq, while the gold wire arrays with 16.7 μm period have a wavelength integrated transmission of 96.4%, with a measured grid sheet resistance of 2.5 Ω/sq. Both results compare favourably with the state of the art^4^.

To assess the technological relevance of the effect here described it is necessary to consider both electrical and optical energy loss mechanisms. In Fig. 3 we present calculations of the total power loss including resistance losses as a function of wire width following the work of Moore^25^, without inclusion of optical cloaking effects. Calculation parameters correspond to a typical concentrator solar cell: emitter sheet resistance of 300 Ω/sq, 500 suns concentration, 1 mm^2^ active area. Fortunately, wire widths of the order of 500 nm are optimal not only from the point of view of maximum optical cloaking efficiency (Fig. 1c), but also from the point of view of minimum series resistance losses (Fig. 3), as well as being above the threshold for problematic effects such as electro-migration^26^, and increased resistivity due to the wire dimensions approaching the electron mean free path length^27^. The minimum in Fig. 3 is due to the cross over between geometries dominated by spreading resistance losses (at large wire spacings), geometries dominated by grid resistance losses (at small wire widths and large wire spacings), and geometries dominated by shadow losses (at large wire widths and small wire spacings)^25^. Contact lines in concentrator solar cells typically have wire spacings of the order of 100 μm, but still suboptimal wire widths of the order of a few μm due to a number of technological difficulties that we have made progress in overcoming (see methods section). The devices that we have fabricated for the purpose of experimentally demonstrating contact cloaking have purposely small wire spacings as otherwise shadow losses would become too small to measure accurately. As the wire spacing is increased, the grid sheet resistance will increase linearly with the spacing, while the shadow losses (the main loss mechanism in our devices) will decrease inversely with the spacing. The spectrally integrated cloaking effect shown in Fig. 1c is independent of wire spacing (see supplementary material), except for very small spacings of the order of the light wavelength, where reduced reflection can be expected due to diffraction suppresion^28^,^29^,^30^.

Using nanofabrication techniques, and with proper optimization, there is ample room to reduce the losses caused by the top contact. In applications making use of a very wide range of wavelengths (such as multijunction solar cells), maximum transmission efficiency is achieved with wire dimensions corresponding to the transition between extinction dominated by resonances and extinction dominated by non-resonant Rayleigh scattering. These results should lead to significant improvements over current designs for concentrator solar cells, light emitting diodes, and flat panel displays.

## Methods

FDTD simulations were performed using Lumerical FDTD Solutions software. The typical line width, height and geometric shadow factor used in concentrator solar cells are 3 μm, 600 nm and 3% respectively^10^. Using this standard design as a starting point, we have explored designs with periods and wire widths an order of magnitude smaller but similar geometrical shadow factors while leaving the grid resistance almost unchanged. The FDTD simulation cell has been designed in the following way:

-The above and below boundaries conditions are set as 12 Perfect Matching Layers (PML) so that all the energy escaping the cell is absorbed without spurious reflections. The cell height has been kept constant at 8 μm for all the simulations, with boundaries 6 μm above the GaAs/air (or AlInP) interface and 2 μm into the GaAs.

-Left and right boundaries are changed depending on the simulation type. When a grid is studied, Periodic Boundary Conditions are used, and when studying an isolated wire 12 PML are used. Isolated wires are studied in a 30 μm wide cell in order to collect light scattered at shallow angles (this is the cause for the observed diffraction effects in the isolated wire shadow efficiency map). For the rest of simulations cell width is changed accordingly with the fixed shadow factor or period requirements.

-For Solar Cell structure simulations, layers are stacked over the GaAs as follows from bottom to top: 20 nm AlInP, 30 nm ZnS, 45 nm Si_3_N_4_. The structure under the metal lines is, from bottom to top 20 nm AlInP, 80 nm contact GaAs layer and the metal (typically 600 nm height). The semi-elliptical shape was chosen due to the results of preliminary tests showing higher cloaking efficiencies than for rectangular cross sections. The incident medium is SiO_2_.

-Materials refraction indexes are taken from the Lumerical materials library corresponding to Palik when possible (GaAs, Ag, SiO_2_, ZnS) and from other databases when not (AlInP:SOPRA, Si_3_N_4_: T.Baak, Au: Johnson and Christy) and are fitted to a single polynomial for the whole spectrum of the source.

-The light source is a plane wave spanning wavelengths from 350 nm to 1600 nm normally incident on the structure from 5 μm over the GaAs interface. Each polarization is simulated separately.

-Charge carrier transport effects are expected to play a very minor role as the changes in the photogeneration profile as a function of depth are small in all studied cases, resulting in negligible changes in the internal quantum efficiency.

-In order to make comparable power transmission measurements between the reference and metal line simulations, meshing has been forced to a 4 nm size in the ARC and metal line area. The 4 nm meshing was chosen after convergence testing.

-In the same fashion the transmission power monitors have been set to a 4 nm height over the GaAs into the AlInP, as some of the carriers generated in the window layer can be collected and contribute to the photocurrent.

-The reflection power monitor is located 500 nm over the source to avoid source influence.

-To calculate the absorption we assume that the sum of reflection, absorption and transmission equals the incident power. This is only approximately true in the calculations using PMLs at the lateral boundaries, and thus absorption could not be calculated reliably in this case.

Diffraction features are barely noticeable in large period arrays, but in the case of short period arrays the diffraction features obscure other spectral features of interest (Rayleigh extinction and localized plasmon resonances). Fortunately these diffraction features in the spectra are very sensitive to the light incidence angle. Because the experiments are done with focused light using a lens with 0.4 numerical aperture, light incidence angles range from 0° to 23° relative to the surface normal, blurring all diffraction features. Simulated spectra for short period arrays have thus also been averaged for incidence angles in the same range as in the experiment. In these simulations the reflection monitor had to be positioned above the light source to avoid artefacts related to the light injection method, and phase preserving periodic boundary conditions had to be used (Bloch periodic boundary conditions).

**Figure 4:**
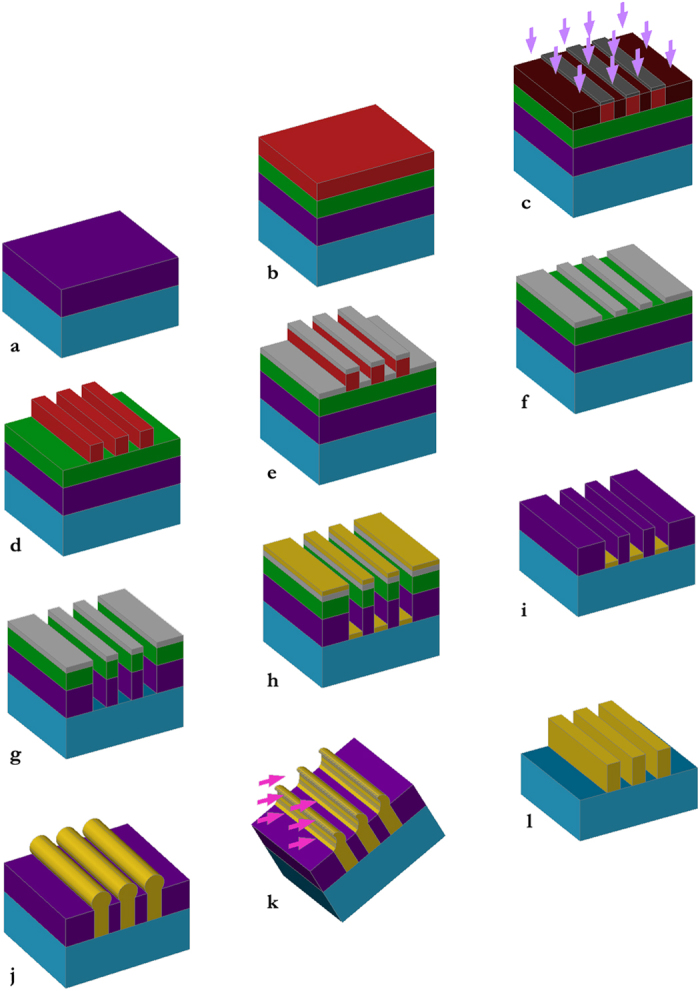
Metal nanowire array fabrication sequence. (**a**) SiOx deposition by CVD. (**b**) Organic antireflective coating and photoresist spin coating. (**c**) Photoresist exposure by laser interference lithography. (**d**) Photoresist development. (**e**) Cr deposition. (**f**) Lift off. (**g**) Reactive ion etching. (**h**) Seed contact metal deposition by sputtering. (**i**) Organic antireflective coating lift off. (**j**) Electrodeposition. (**k**) Grazing angle Ar ion milling. (**l**) Finished contact array.

All solar cells were fabricated on GaAs *n*-type substrates by solid source molecular beam epitaxy (MBE). The fabrication procedure is summarized in Fig. 4. Devices were processed using a combination of UV lithography techniques, plasma etching and a double step metallization. The first step (Fig. 4a) involves plasma enhanced chemical vapor deposition (PECVD) to deposit a thick sacrificial layer of SiO_x_ (400–600 nm) to both protect the semiconductor and to serve as a mold for the electrodeposition. A 160 nm thick layer of iCON-16 organic bottom antireflective coating (BARC) was deposited by spin coating and prebaked at 90 °C for 90 s. This layer had three functions: to planarize the surface prior to resist coating; to lead to a better resolution by preventing reflection and standing waves in the photoresist; and to serve as a lift-off layer after the contact seed layer deposition. On top of the BARC, a 500 nm thick layer of photoresist was spun-on (Fig. 4b). For the short period Ag arrays, patterns were defined by laser interference lithography (LIL) on NR9-500PY negative photoresist after prebaking at 120 °C for 120s, whereas for the long period Au arrays contact mask lithography on S1805 positive photoresist was used after prebaking at 115 °C for 60 s. For LIL patterning, a Lloyd’s mirror interferometric lithography system was used^31^ with a 355-nm third harmonic YAG laser source; the power density was adjusted so that a dose of ~30 mJ/cm^2^ was delivered in about 4000 pulses at 80 Hz. After a post-exposure bake, the patterns were developed in a KOH based developer. The resulting patterns consisted of 400–600 nm wide lines over large areas (>1 cm^2^). For long period Au arrays, photoresist development was followed by Cr deposition as a mask for dry etching and photoresist lift off (Fig. 4f), whereas for short period Ag arrays, the photoresist was directly used as a dry etching mask. Reactive ion etching (RIE) in a Oxford Plasmalab 80 by means of a CHF_3_ plasma was then performed to transfer the line pattern to the device surface through the iCON-16 and photoresist/SiO_x_ layers. For the Ag short period arrays, details of this plasma etching process include: chamber pressure, 19 mTorr; RIE power, 100 W; wafer temperature, 30 °C; output DC Bias, ~275 V. This etch was stopped with a thin layer (20 nm) of SiO_x_ at the bottom of the trench to protect the GaAs. In the gold wire arrays, verticality of the side walls was improved by using a pulsed etch with an on/off cycle of 30 s/120 s, with N_2_ flow in the first off minute, as well as using a lower pressure (5 mTorr), higher power plasma (200 W, 475V). A quick dilute HF dip was used to remove the remaining SiO_x_ at the bottom of the trenches and the bus bars. For the Au wires, an ohmic contact was formed by deposition of a thin layer of metal that also served as a seed layer for the electrodeposition (Fig. 4i). The metallization was thermally evaporated Cr/Au (10/60 nm). Silver wires did not require a seed layer. A lift-off of the iCON-16 BARC layer and the metal overlayer was then performed (Fig. 4j). Next, the lines were thickened by a potentiostat controlled electrodeposition step. A cyanide-free gold sulphite/thiosulphate based solution was used without additives. Gold was electrodeposited at a constant potential of −0.3 V vs. Ag/AgCl for 40–60 s using a 0,03 M NaAuCl_4_ aqueous solution kept at 60 °C with 220 rpm agitation. To ensure complete filling we let the lines overflow the trench and then removed the excess material by Ar ion-milling (reactive ion beam etching with a 450 V plasma) at a incidence angle of 65° away from the surface normal (Fig. 4k). Silver was electrodeposited at a constant current density of 0.8 mA/cm^2^ from a 100 ml water based solution with 0.4 g AgK(CN)_2_ + 7 g KCN + 2.5 g K_2_CO_3_. There was no need to remove excess silver as this process allowed for a precise stopping of the electrodeposition after trench filling. In some of the samples, the SiO_x_ layer was etched in diluted HF for SEM inspection, but all samples were inmersed in mineral oil index matched to SiO_x_ for optical characterization. For device separation, mesas were defined by means of photolithography and wet etching of the GaAs with H_3_PO_4_. I-V curves were measured in the dark to characterize the electrical performance. For photocurrent measurements, samples were immersed in high purity mineral oil (tetradecane) to protect silver from tarnishing, as well as to emulate the refractive index of a concentrator secondary stage. To minimize the possible effect of a higher series resistance in the reference experiments, the photocurrent measurements were done at low photon fluxes (of the order of 1 nW). The AC photocurrent generated by light modulated at 477 Hz was collected through 20 μm diameter Tungsten probes and demodulated using a lock-in amplifier. The light from a halogen lamp was dispersed by a 0.3 m focal length monochromator set to a spectral resolution of ~1 nm and then coupled into a 200 micron core size multimode optical fibre. Light coming from the fibre was recollimated and linearly polarized parallel or perpendicular to the grid direction using a fixed Glan-Thompson polarizer and an achromatic half wave retarder. The polarized beam was then focused onto the device within a 30 μm diameter spot using a low magnification objective lens (NA = 0.4). A filter was required to exclude high energy photons due to higher diffraction orders in measurements across the 550 nm to 850 nm spectral range.

The experimental shadow efficiencies are obtained as *S* = (*I*_*0*_−*I*)/*I*_*0*_, where *I* and *I*_*0*_ are the currents measured in areas with and without nanowires within the same device under very low intensity illumination (1 nW) and an illumination spot focused to a diameter of 30 μm. The reported transmission values are obtained by integrating 1−*S* in wavelength. The illumination spot was scanned over the sample to ensure that the devices were homogenous and local features did not affect the spectra. Spectra were recorded for the two linear polarizations changing only the retarder angle. The grid sheet resistance was measured by the four point probe method with a high precision current source and a separate nanovoltmeter.

## Additional Information

**How to cite this article**: San Román, E. *et al*. Cloaking of solar cell contacts at the onset of Rayleigh scattering. *Sci. Rep*. **6**, 28669; doi: 10.1038/srep28669 (2016).

## Supplementary Material

Supplementary Information

Supplementary Movie S1

## Figures and Tables

**Figure 1:**
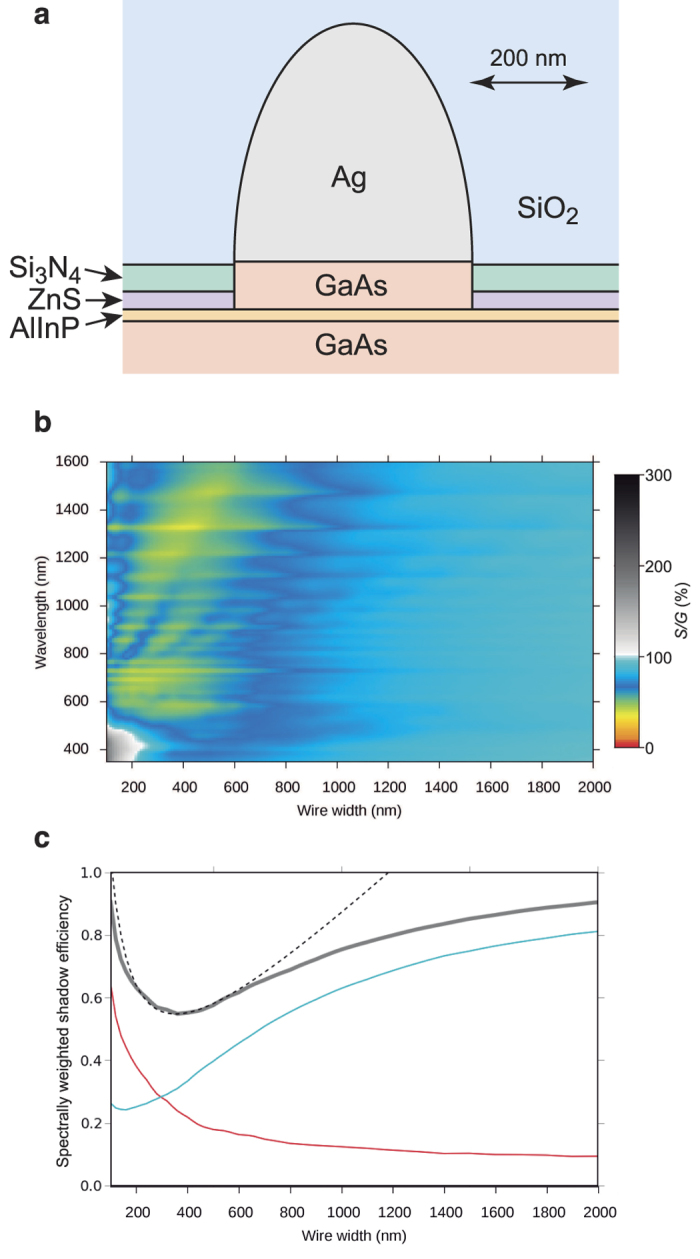
Shadow efficiency for silver wires on a GaAs solar cell. (**a**) Structure used to calculate the optical losses caused by the top metal contact. (**b**) The fraction of the incoming power lost due to reflection and absorption at the metal contact is plotted normalized to the fraction of the area taken up by the contact as a function of wavelength and wire width. The wire height and period are fixed at 600 nm and 10 μm, respectively. Normal incidence illumination with unpolarized light. (**c**) Spectrally integrated shadow efficiency as a function of wire width. The data are weighted with the direct+ circumsolar AM1.5 solar spectrum^18^. The red and blue lines represent, respectively, the absorption and reflection contributions to the total shadow (thick grey line). The dashed line is a fit to an *ax + b/x* expression with only two free parameters were the two terms can be identified with non-resonant Rayleigh extinction and resonant extinction, respectively.

**Figure 2:**
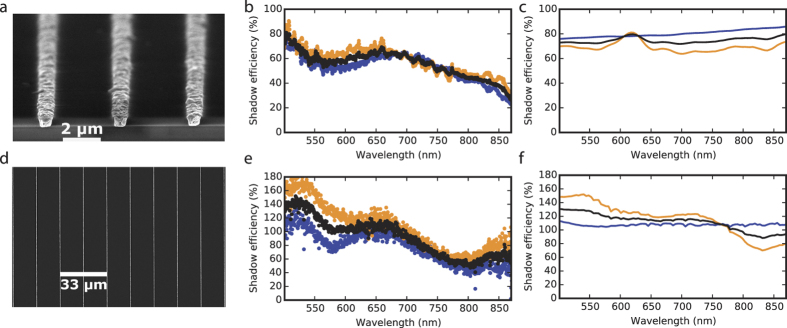
Comparison of experimental and simulated results for silver and gold wire arrays. (**a**) Scanning electron microscopy image of silver wires electrodeposited through a SiO_x_ mask on a GaAs solar cell. The length of the scale bar is half the 4 μm array period. (**b**) Experimentally measured shadow efficiencies for the silver wires in Fig. 2a. Results for transversal polarization, parallel polarization, and unpolarized light are shown in orange, blue, and black, respectively. (**c**) FDTD simulations corresponding to the experimental results for silver wires in Fig. 2b. (**d**) Scanning electron microscopy image of gold wires electrodeposited through a SiO_x_ mask on a GaAs solar cell. The length of the scale bar is twice the 16.7 μm array period. (**e**) Experimentally measured shadow efficiencies for gold wires. (**f**) FDTD simulations corresponding to the experimental results for gold wires.

**Figure 3:**
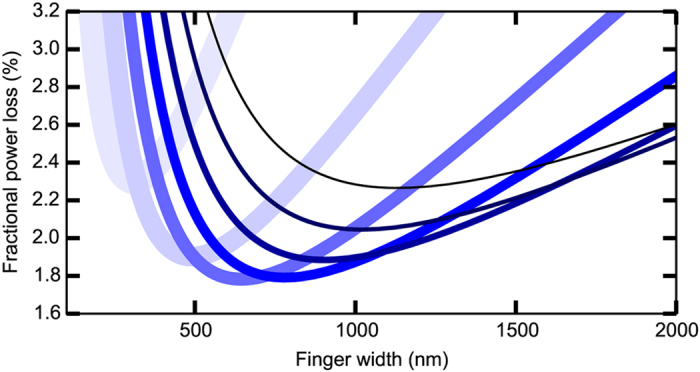
Calculated power loss caused by the top contact. The plot represents the ratio of the power loss to the device power output. Lighter to darker shades of blue correspond respectively to array periods of 20, 40, 60, 80, 100, 120 and 140 μm.

## References

[b1] Meulenberg A. The sawtooth coverslide-A new means of coupling light into solar cells. J. Energy 1, 151 (1977).

[b2] Schumann M. F. . Cloaked contact grids on solar cells by coordinate transformations: designs and prototypes. Optica 2, 850 (2015).

[b3] Hsu P.-C. . Performance enhancement of metal nanowire transparent conducting electrodes by mesoscale metal wires. Nature Comm. 4, 2114 (2013). 10.1038/ncomms3522 24065116

[b4] Wu H. . A transparent electrode based on a metal nanotrough network. Nature Nanotech. 8, 421–425 (2013). 10.1038/nnano.2013.84 23685985

[b5] Van Beijnum F. . Quasi-cylindrical wave in expericontribution ments on extraordinary optical transmission. Nature 492, 411 (2012). 23257884 10.1038/nature11669

[b6] Ebbesen T. W., Lezec H. J., Ghaemi . Extraordinary optical transmission through sub-wavelength hole arrays. Nature 391, 667–669 (1998).

[b7] Wu C. C., Wu C. I., Sturm J. C. & Kahn A. Surface modification of indium tin oxide by plasma treatment: An effective method to improve the efficiency, brightness, and reliability of organic light emitting devices. Appl. Phys. Lett. 70, 1348 (1997).

[b8] Cai M. . Extremely Efficient Indium–Tin-Oxide-Free Green Phosphorescent Organic Light-Emitting Diodes. Adv. Mater. 24, 4337 (2012). 22786793 10.1002/adma.201202035

[b9] Van de Groep J., Spinelli P. & Polman A. Transparent Conducting Silver Nanowire Networks. Nano Lett. 12, 3138 (2012). 22554260 10.1021/nl301045a

[b10] García I., Rey-Stolle I., Galiana B. & Algora C. A 32.6% efficient lattice-matched dual-junction solar cell working at 1000 suns. Appl. Phys. Lett. 94, 053509 (2009).

[b11] Philipps S., Dimroth F. & Bett A. High Efficiency III-V Multi-Junction Solar Cells. In: McEvoy A. J., Castaner L., Markvart T. , Practical Handbook of Photovoltaics Amsterdam, Boston: Elsevier Academic Press, (2012).

[b12] Atwater H. A. & Polman A. Plasmonics for improved photovoltaic devices. Nature Mater. 9, 205–213 (2010). 20168344 10.1038/nmat2629

[b13] Catchpole K. R. & Polman A. Design principles for particle plasmon enhanced solar cells. Appl. Phys. Lett. 93, 191113 (2008).

[b14] Catrysse P. B. & Fan S. Nanopatterned Metallic Films for Use As Transparent Conductive Electrodes in Optoelectronic Devices. Nano Lett. 10, 2944 (2010). 20698607 10.1021/nl1011239

[b15] Bohren C. F. & Huffman D. R. Absorption and Scattering of Light by Small Particles (Wiley, New York, 1983).

[b16] Luk’yanchuk B. S. & Ternovsky V. Light scattering by a thin wire with a surface-plasmon resonance: Bifurcations of the Poynting vector field. Phys. Rev. B 73, 235432 (2006).

[b17] Newton R. G. Optical Theorem and Beyond. Am. J. Phys 44, 639–642 (1976).

[b18] ASTM-G173, *Standard Tables for Reference Solar Spectral Irradiances*, American Society for Testing and Materials, 2008.

[b19] Yang Z. J. . Ultimate Limit of Light Extinction by Nanophotonic Structures. Nano Lett. 15, 7633–7638 (2015). 26478949 10.1021/acs.nanolett.5b03512

[b20] Knight M. . Nanoparticle-Mediated Coupling of Light into a Nanowire. Nano Lett. 7, 2346 (2007). 17629348 10.1021/nl071001t

[b21] Ditlbacher H. . Silver Nanowires as Surface Plasmon Resonators. Phys. Rev. Lett. 95, 257403 (2005). 16384506 10.1103/PhysRevLett.95.257403

[b22] Shao L. . Comparison of the plasmonic performances between lithographically fabricated and chemically grown gold nanorods. Phys. Chem. Chem. Phys. 17, 10861 (2015). 25820223 10.1039/c5cp00715a

[b23] Li Z. . Effect of a proximal substrate on plasmon propagation in silver nanowires. Phys. Rev. B 82, 241402R (2010).

[b24] Billaudeau C. . Tailoring radiative and non-radiative losses of thin nanostructured plasmonic waveguides. Optics Express 17(5), 3490 (2009). 19259187 10.1364/oe.17.003490

[b25] Moore A. R. An Optimized Grid Design for a Sun-Concentrator Solar Cell. RCA Review, Vol. 40, June 1979, P. 140–152. 40 (June 1, 1979): 140–52.

[b26] Brillson L. J. Contacts to Semiconductors: Fundamentals and Technology. Noyes, 1993.

[b27] Marom H., Mullin J. & Eizenberg M. Size-dependent resistivity of nanometric copper wires. Phys. Rev. B 74, 045411 (2006).

[b28] Yu Z., Aaswath A. & Fan S. Fundamental limit of nanophotonic light trapping in solar cells. Proceedings of the National Academy of Sciences 107, 17491–17496 (2010). 10.1073/pnas.1008296107 PMC2955111 20876131

[b29] Buencuerpo J., Llorens J. M., Dotor M. L. & Ripalda J. M. Photon management with nanostructures on concentrator solar cells. Appl. Phys. Lett. 103, 083901 (2013).

[b30] San Román E. . High transmission nanowire contact arrays with subwavelength spacing, Phys. Status Solidi RRL, 1–4 (2015)/doi: 10.1002/pssr.201510367

[b31] Brueck S. R. J. Optical and Interferometric Lithography: Nanoscale Enablers. Proc. IEEE 93, 1704–1721 (2005).

