# Supplementary material for: Cloaking of solar cell contacts at the onset of Rayleigh scattering
Source: Sci Rep. 2016 Jun 24;6:28669. doi: 10.1038/srep28669 (PMC4919638; doi:10.1038/srep28669)
Supplement: Supplementary Information [file srep28669-s1.pdf]

Supplementary Information

Cloaking of solar cell contacts at the onset of Rayleigh scattering

Etor San Román[1], Alan Vitrey[1], Jerónimo Buencuerpo[1], Iván Prieto[1], José M. Llorens[1],

Antonio García-Martín[1], Benito Alén[1], Anabil Chaudhuri,[2] Alexander Neumann,[2]

S. R. J. Brueck[2], José M. Ripalda[1,*]

[1]Instituto de Microelectrónica de Madrid (IMM – CNM - CSIC),
Isaac Newton 8, PTM, E-28760 Tres Cantos, Madrid, Spain

[2]Center for High Technology Materials, University of New Mexico, Albuquerque, NM 87106, USA

[1] IMM-Instituto de Microelectrónica de Madrid (CNM-CSIC), Isaac Newton 8, PTM, E-28760 Tres Cantos, Madrid, Spain

[2] Center for High Technology Materials, University of New Mexico, Albuquerque, NM 87106, USA

Contents:

Extinction efficiencies for free standing cylinders 3
Main resonances of a silver wire in a ZnS matrix 4
Scattering and absorption efficiencies for Silver in ZnS 6
Scattering and absorption efficiencies for Aluminium in SiO_2 8
Absorption efficiency as a function of matrix material 10

FDTD simulations 11
Reflection and absorption efficiencies for silver wires on a GaAs substrate 11
Silver wires on a solar cell. Fixed periodicity 15
Silver wires on a solar cell. Fixed geometric shadow. 18
Silver wires on a solar cell. Isolated wires 21
Aluminium wires on a solar cell. Fixed geometric shadow 23
Video: Steady state near field as a function of wavelength. 26

Device characterization 27
Quantum efficiency 27
IV curves 27

Extinction efficiencies for free standing cylinders

To better understand the basic features in the scattering and absorption spectra of metal wires we have used the formalism given by Bohren and Huffman (reference 13 in main text) for a single metal wire in an infinite dielectric matrix. These are far field extinction efficiencies that can be separated in scattering and absorption efficiencies (effective cross sections normalized to the wire projected geometrical area).

Note that in the large size limit, the far field extinction (scattering + absorption) cross section asymptotically tends to twice the geometrical cross section, a surprising but well established fact known as the extinction paradox. This apparent paradox is due to a large contribution to the far field extinction at small scattering angles.

Main resonances of a silver wire in a ZnS matrix

To aid in the interpretation of our results, we separately present here each of the main resonances in an ideal, infinite, isolated metal cylinder.

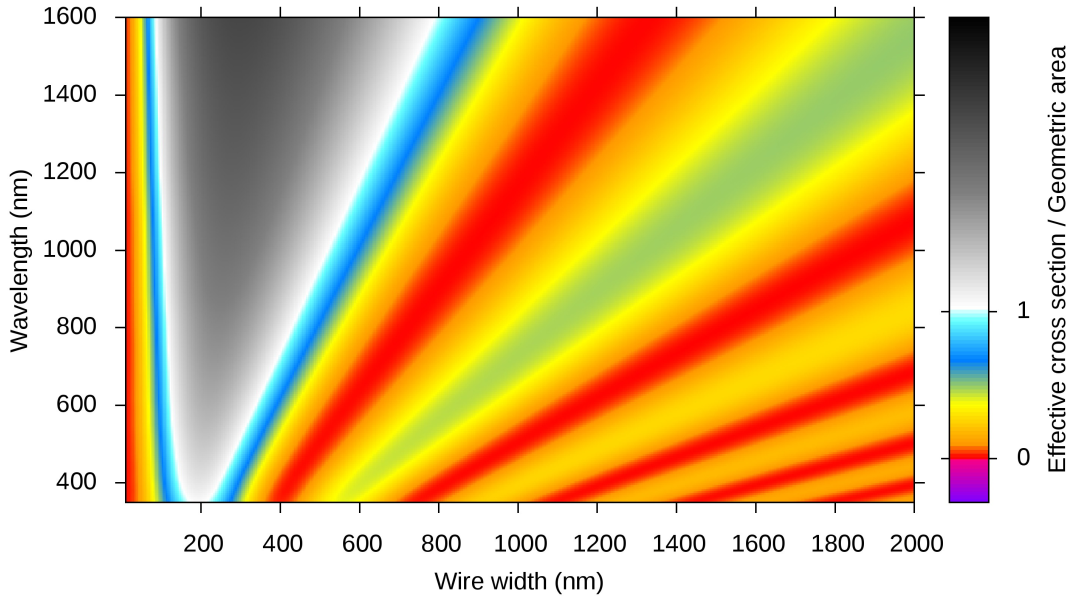

Suppl. Fig. S1. Contribution to the extinction efficiency from the monopole resonance (l=0). **Parallel** polarization.

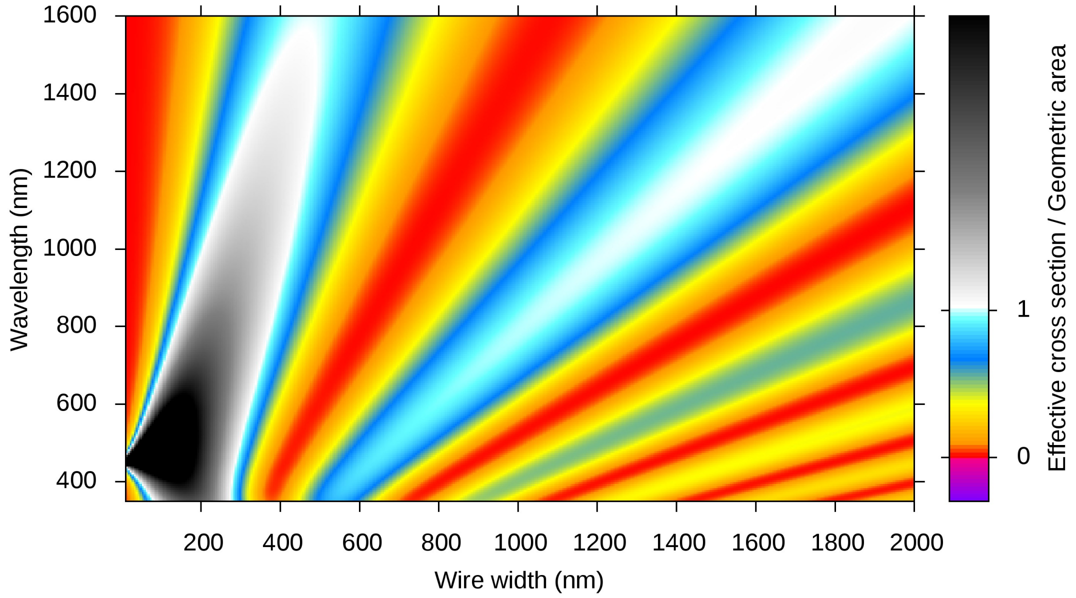

Suppl. Fig. S2. Contribution to the extinction efficiency from the dipole localized surface plasmon resonance (l=1). Transversal polarization.

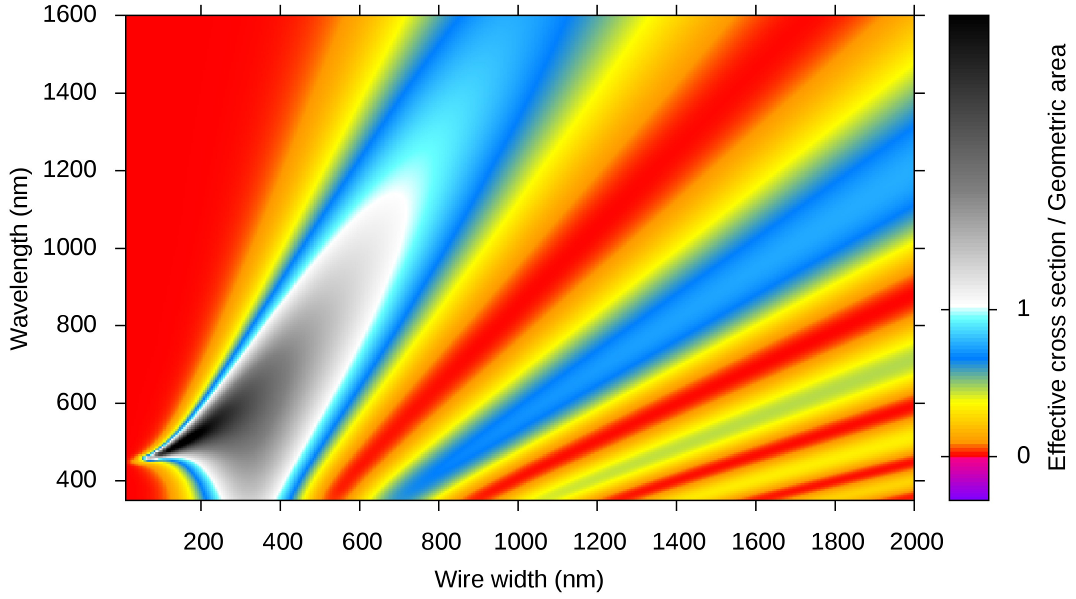

Suppl. Fig. S3. Contribution to the extinction efficiency from the quadrupole localized surface plasmon resonance (l=2). Transversal polarization.

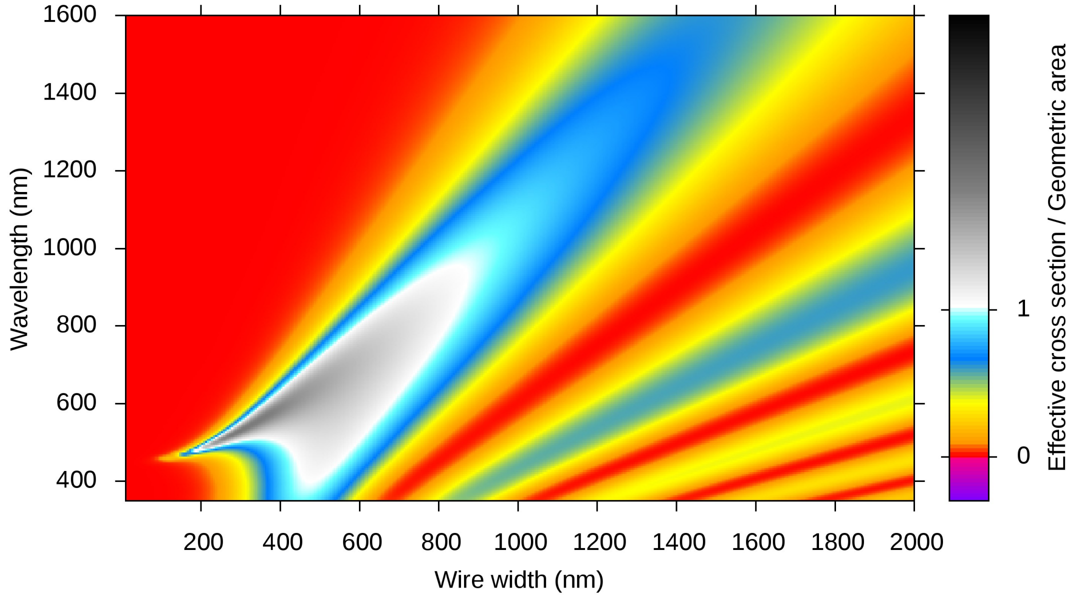

Suppl. Fig. S4. Contribution to the extinction efficiency from the hexapole localized surface plasmon resonance (l=3). Transversal polarization.

Scattering and absorption efficiencies for Silver in ZnS

A summation of the infinite series of resonances presented above results in the following extinction efficiencies.

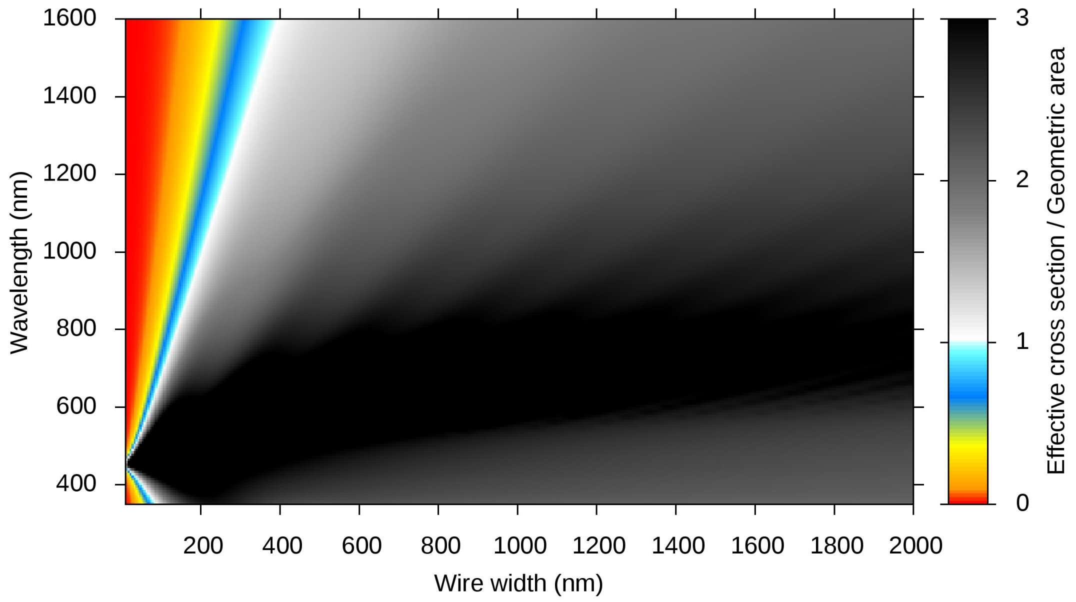

Suppl. Fig. S5. Scattering efficiency for a silver wire in a ZnS matrix. Electric field perpendicular to the wire.

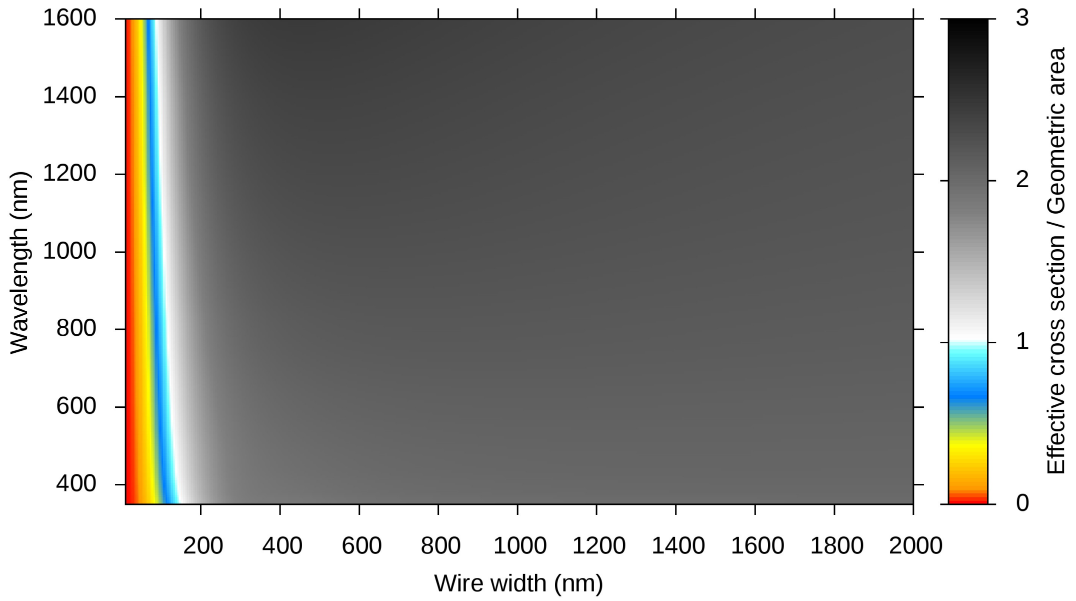

Suppl. Fig. S6. Scattering efficiency for a silver wire in a ZnS matrix. Electric field parallel to the wire.

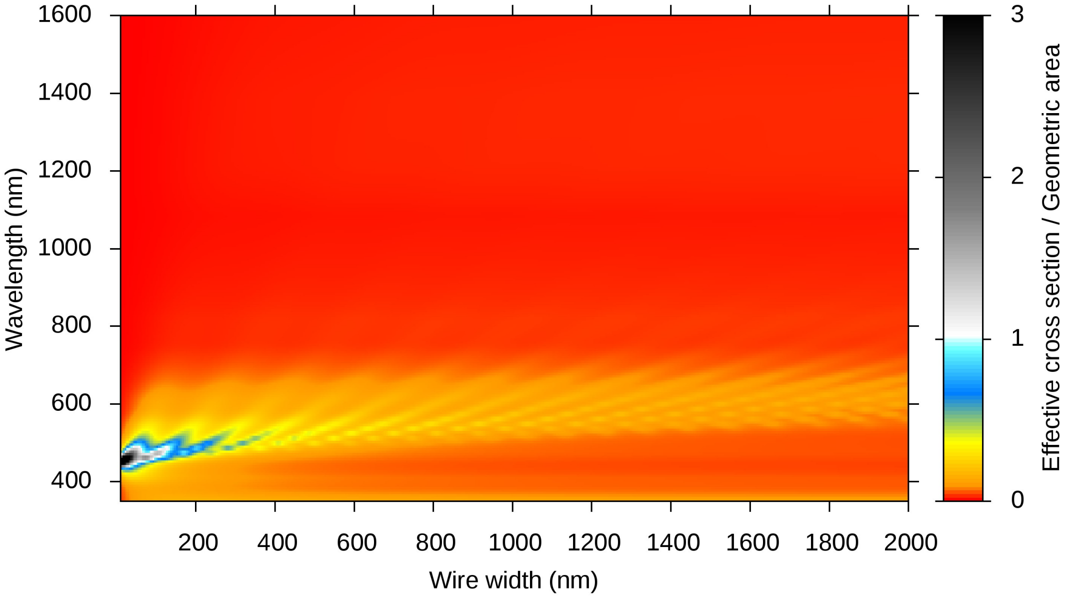

Suppl. Fig. S7. Absorption efficiency for a silver wire in a ZnS matrix. Electric field perpendicular to the wire.

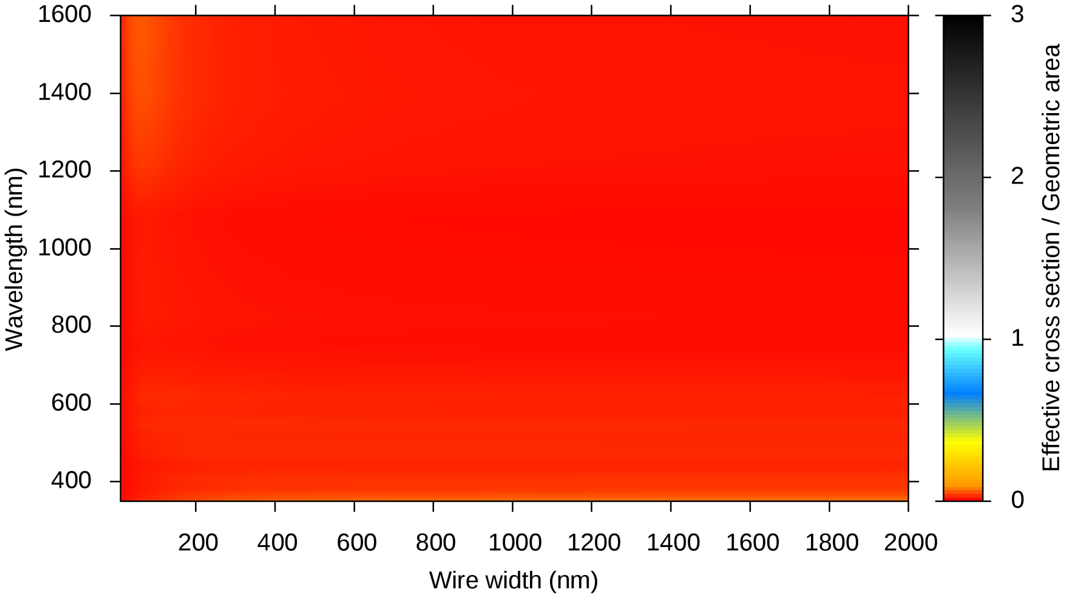

Suppl. Fig. S8. Absorption efficiency for a silver wire in a ZnS matrix. Electric field parallel to the wire.

Scattering and absorption efficiencies for Aluminium in SiO_2

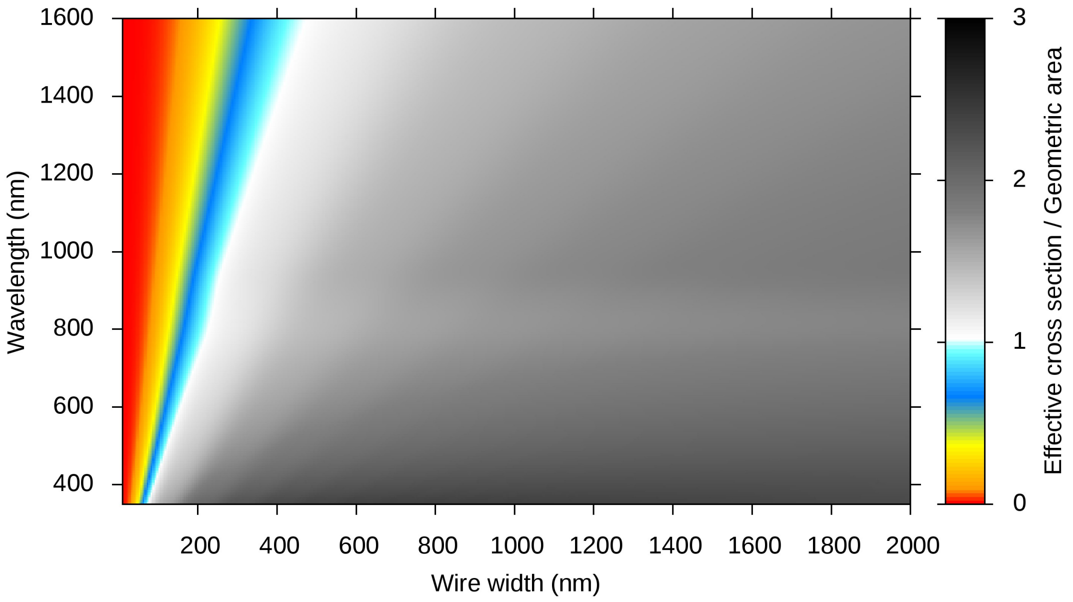

Suppl. Fig. S9. Scattering efficiency for an aluminium wire in a SiO2 matrix. Electric field perpendicular to the wire.

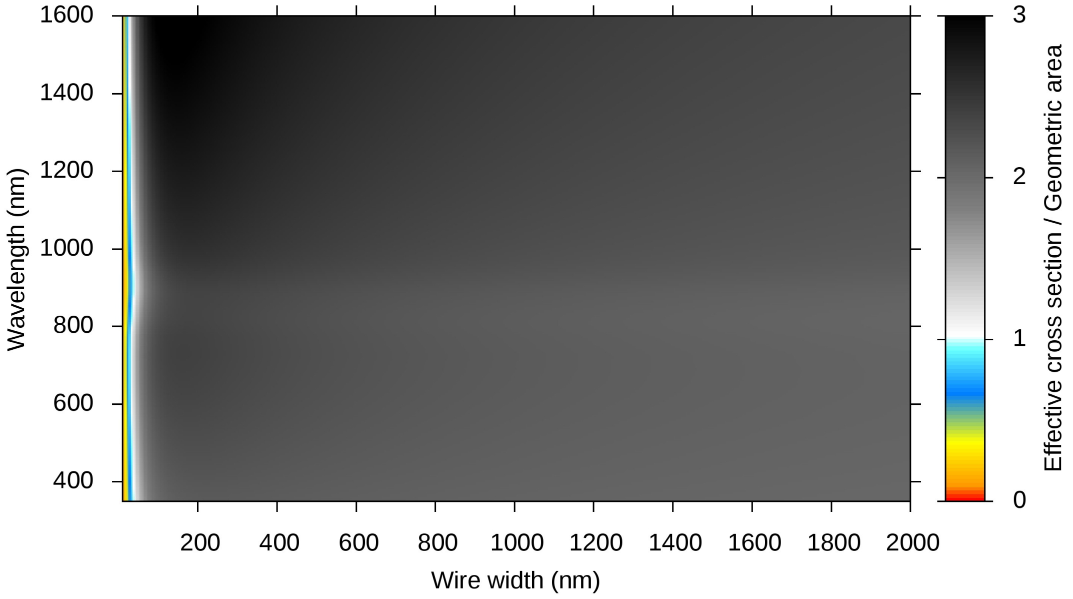

Suppl. Fig. S10. Scattering efficiency for an aluminium wire in a SiO_2 matrix. Electric field parallel to the wire.

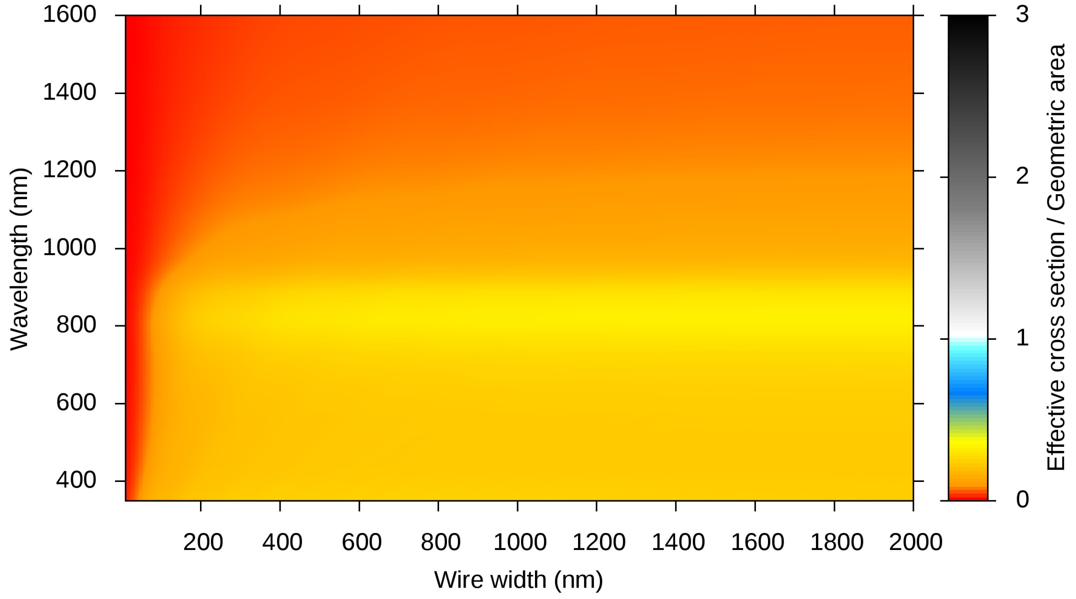

Suppl. Fig. S11. Absorption efficiency for an aluminium wire in a SiO2 matrix. Electric field perpendicular to the wire

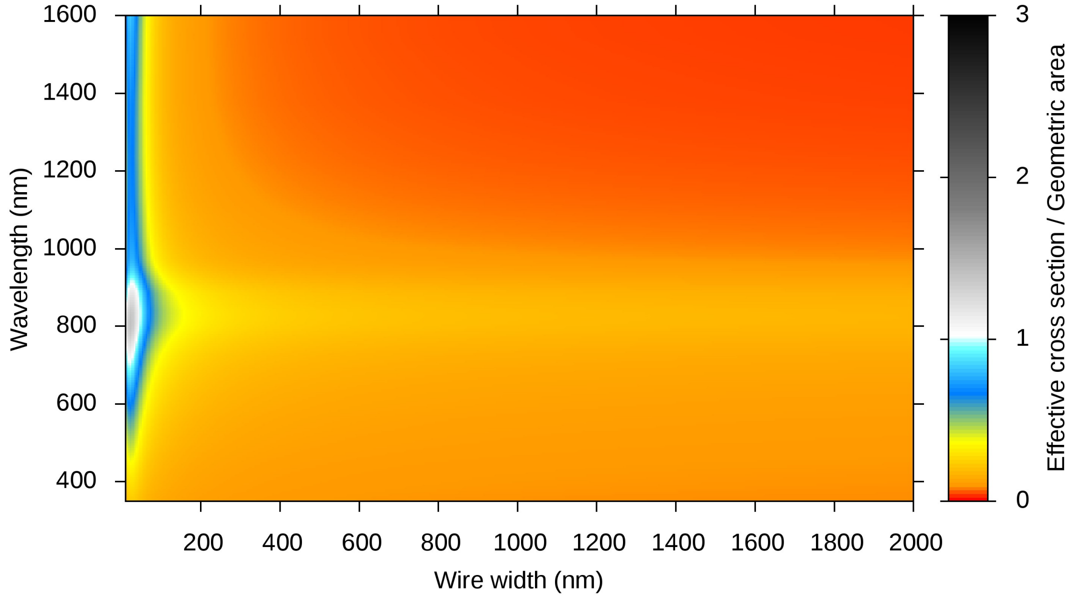

Suppl. Fig. S12. Absorption efficiency for an aluminium wire in a SiO_2 matrix. Electric field parallel to the wire.

Absorption efficiency as a function of matrix material

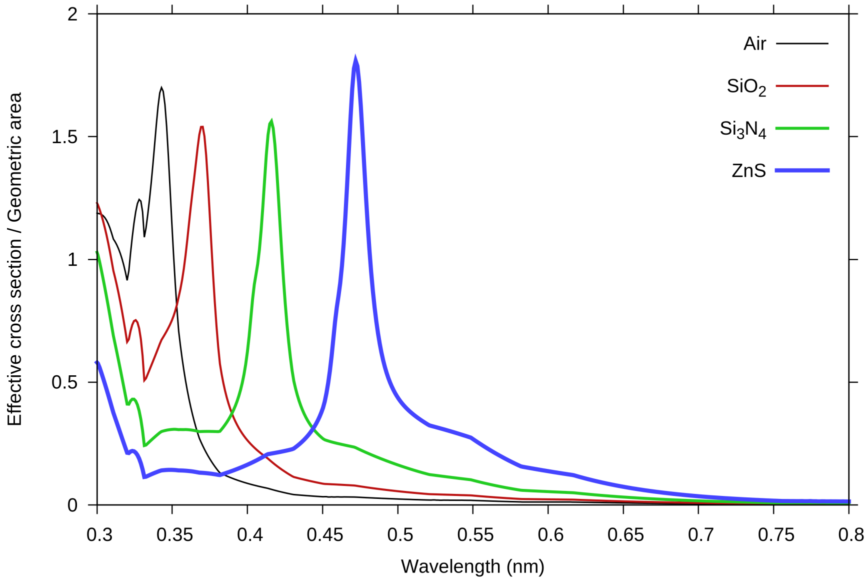

Suppl. Fig. S13. Absorption efficiency as a function of wavelength and matrix material for a silver cylinder with a 100 nm diameter illuminated under normal incidence with transversal polarization.

FDTD simulations

Reflection and absorption efficiencies for silver wires on a GaAs substrate

Wire height = 600 nm.

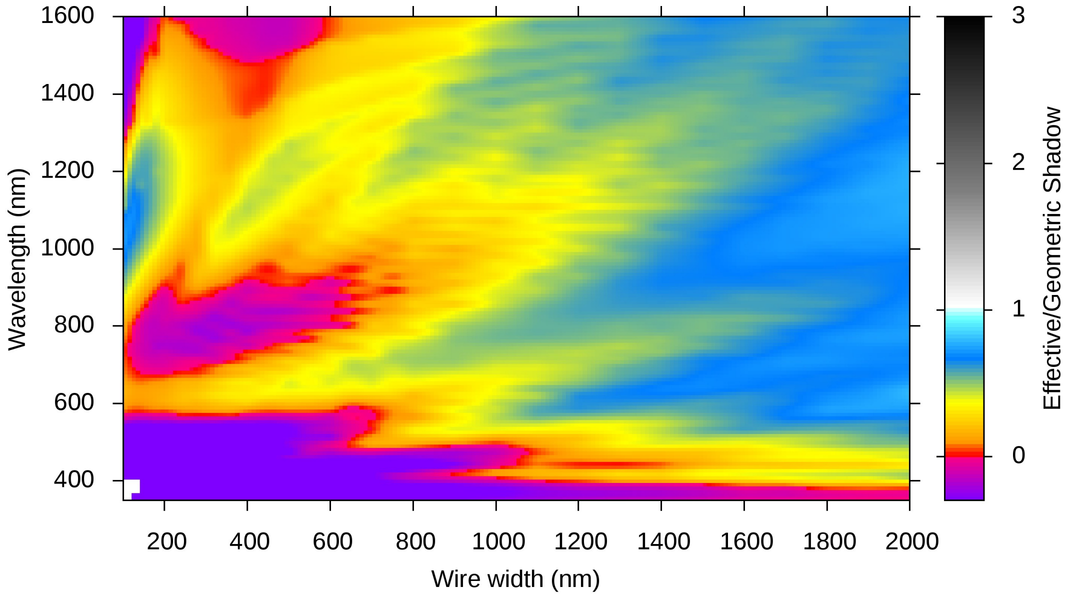

Suppl. Fig. S14. Reflection efficiency for an array of silver wires on a GaAs substrate. Electric field perpendicular to the wire. Periodic boundary conditions with fixed shadow factor (3%).

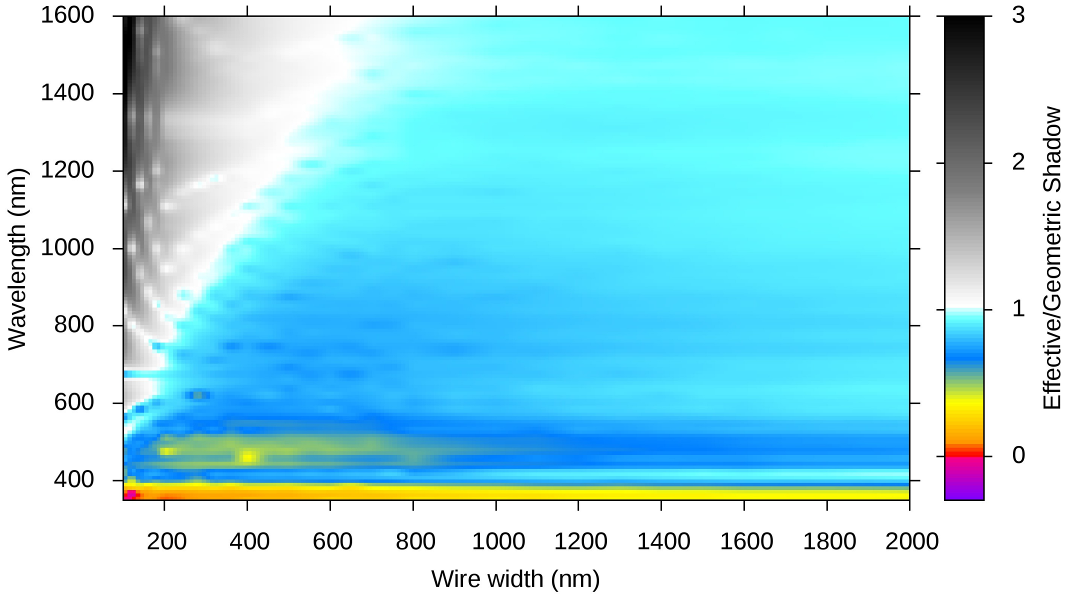

Suppl. Fig. S15. Reflection efficiency for an array of silver wires on a GaAs substrate. Electric field parallel to the wire. Periodic boundary conditions with fixed shadow factor (3%).

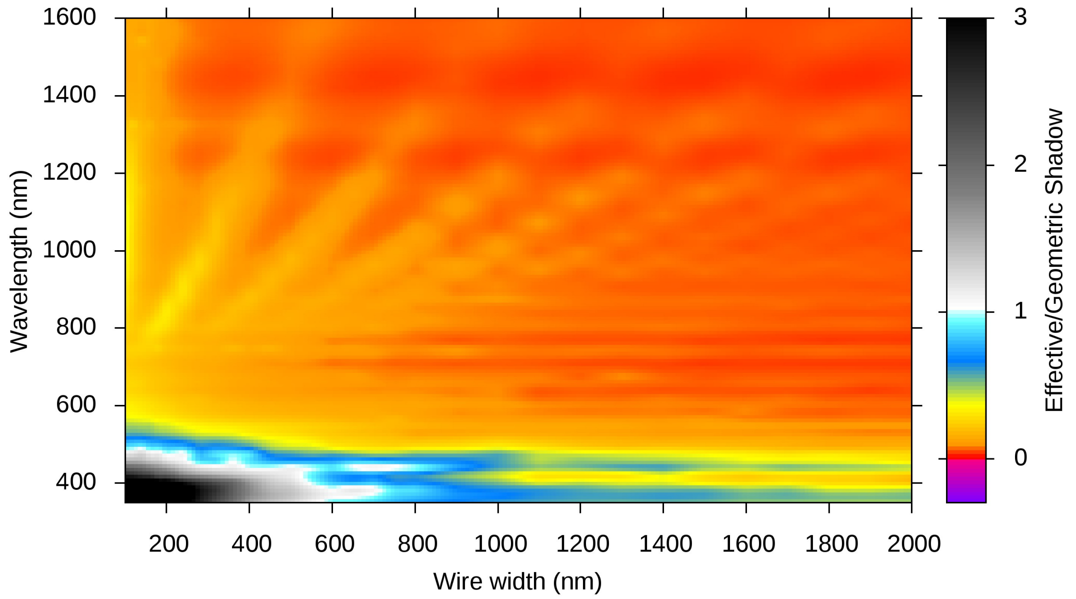

Suppl. Fig. S16. Absorption efficiency for an array of silver wires on a GaAs substrate. Electric field perpendicular to the wire. Periodic boundary conditions with fixed shadow factor (3%).

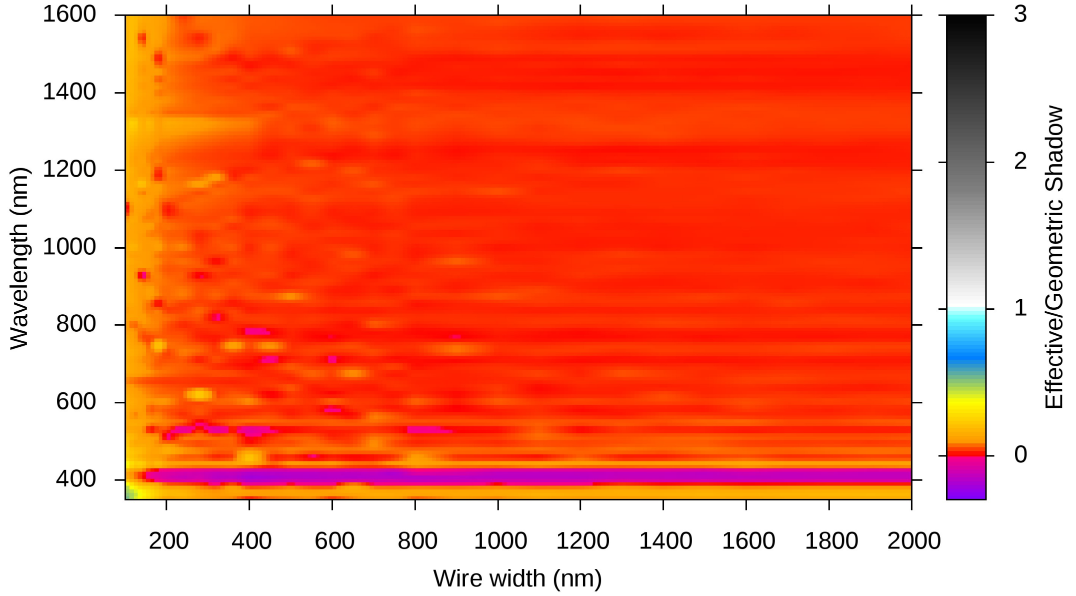

Suppl. Fig. S17. Absorption efficiency for an array of silver wires on a GaAs substrate. Electric field parallel to the wire. Periodic boundary conditions with fixed shadow factor (3%).

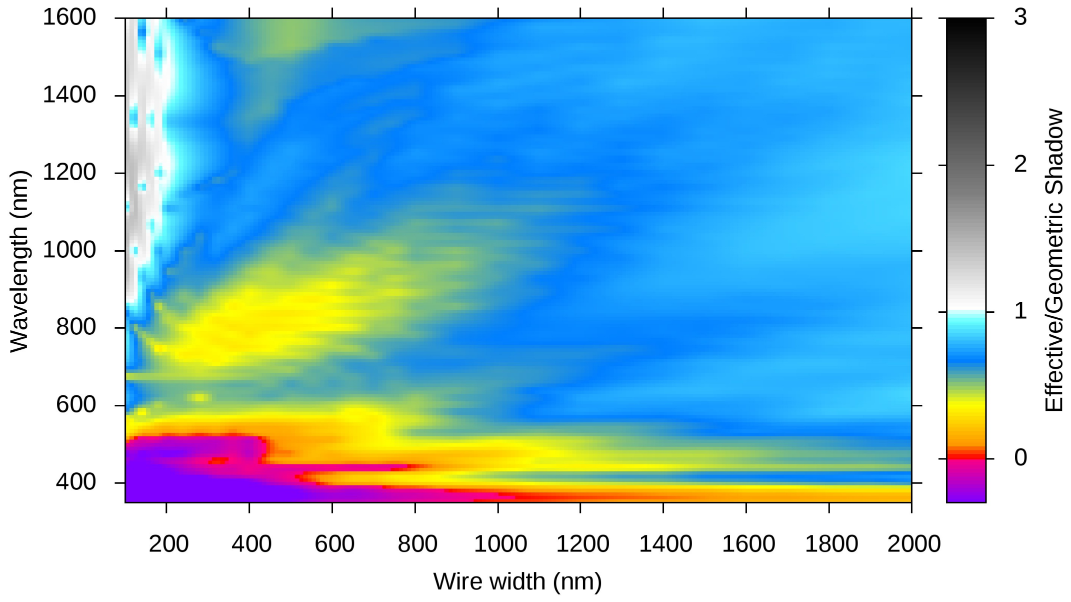

Suppl. Fig. S18. Reflection efficiency for an array of silver wires on a GaAs substrate. Unpolarized light. Periodic boundary conditions with fixed shadow factor (3%).

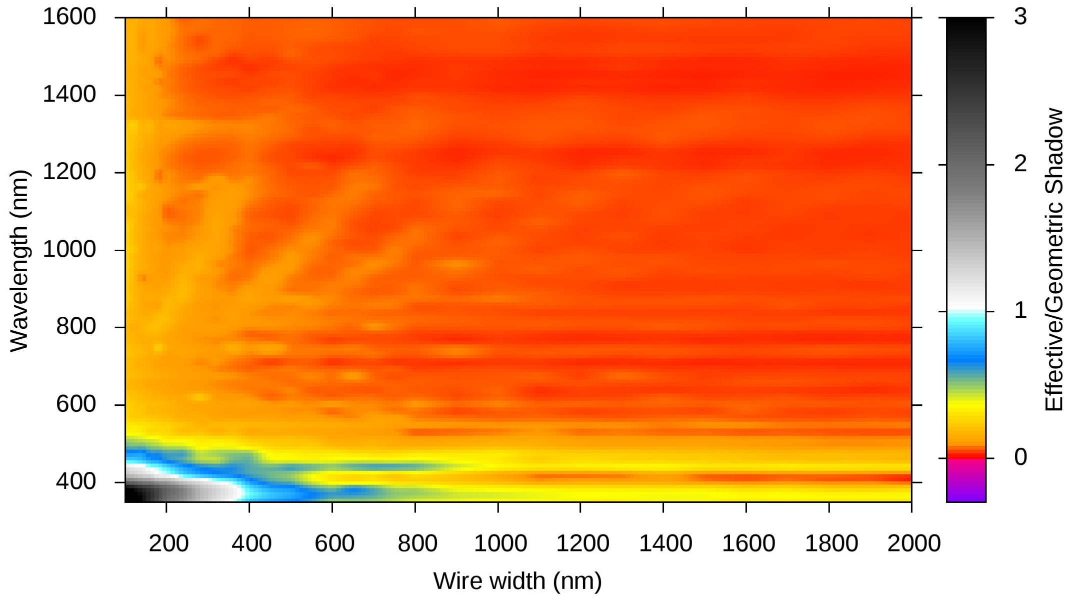

Suppl. Fig. S19. Absorption efficiency for an array of silver wires on a GaAs substrate. Unpolarized light. Periodic boundary conditions with fixed shadow factor (3%).

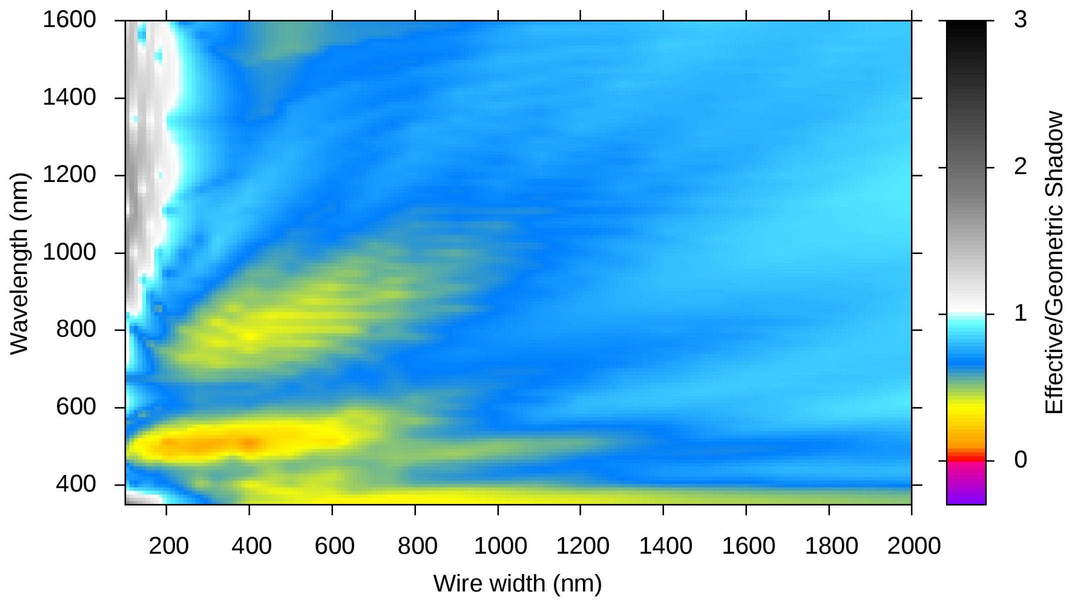

Suppl. Fig. S20. Shadowing efficiency for an array of silver wires on a GaAs substrate. Unpolarized light. Periodic boundary conditions with fixed shadow factor (3%).

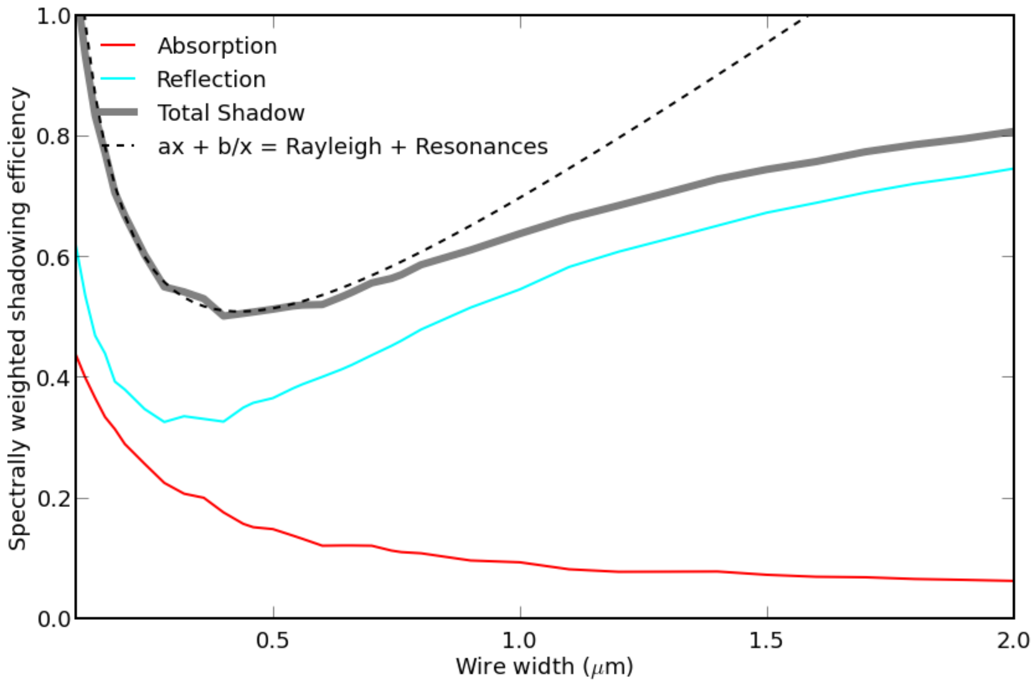

Suppl. Fig. S21. Spectrally weighted shadowing efficiency separated in absorption and reflection contributions for an array of silver wires on a GaAs substrate. The data are weighted with the direct + circumsolar AM1.5 solar spectrum. Periodic boundary conditions with fixed shadow factor (3%).

Silver wires on a solar cell. Fixed periodicity

The corresponding geometry is shown in Fig. 3 of the main text. Wire height = 600 nm.

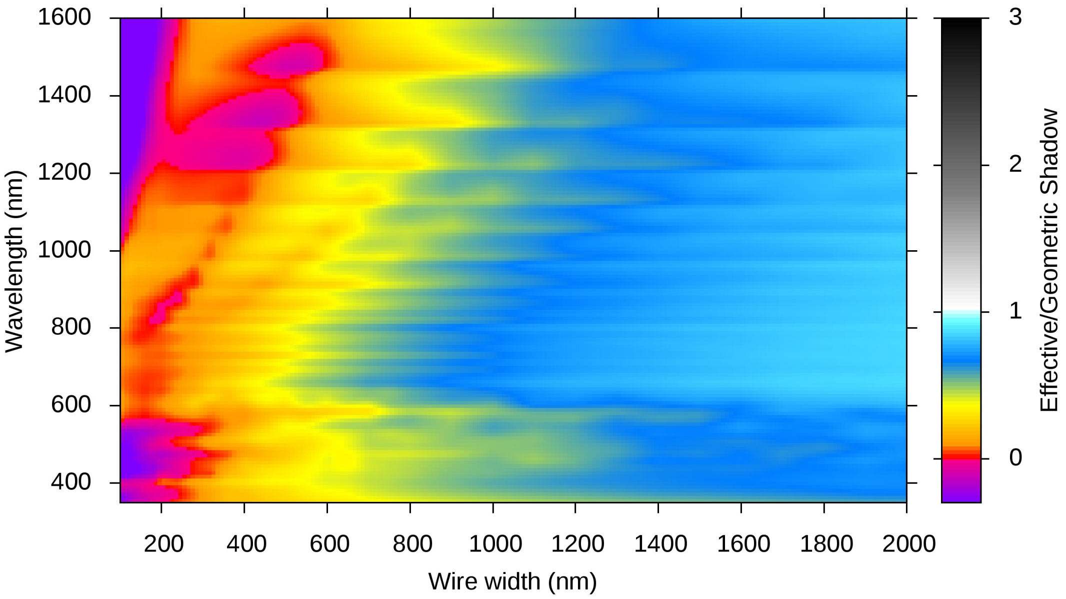

Suppl. Fig. S22. Reflection efficiency for an array of silver wires on a solar cell. Electric field perpendicular to the wire. Periodic boundary conditions with fixed periodicity (10^{-5} m).

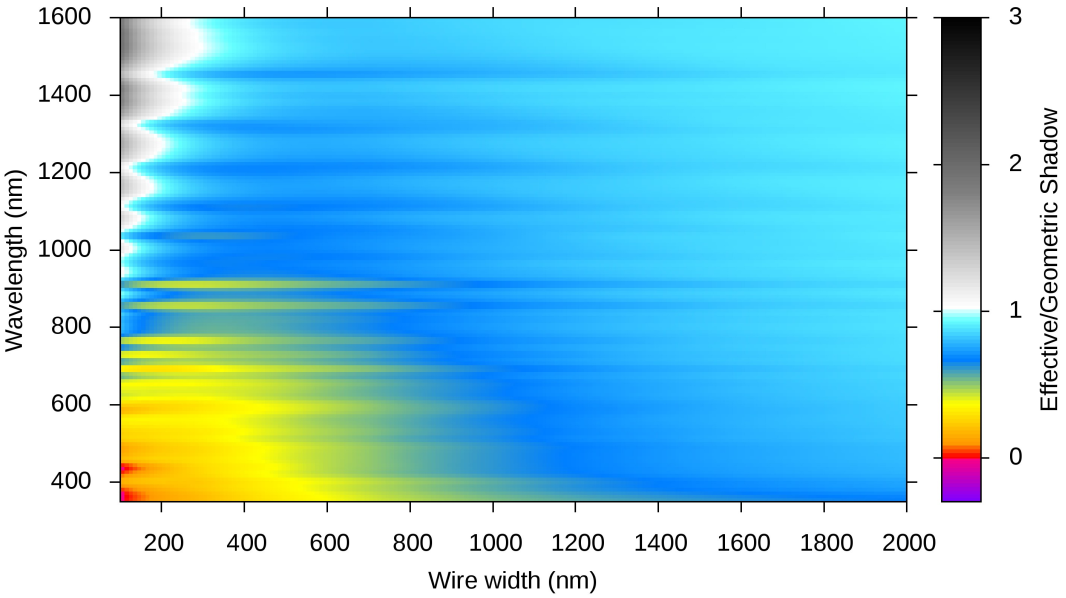

Suppl. Fig. S23. Reflection efficiency for an array of silver wires on a Solar cell. Electric field parallel to the wire. Periodic boundary conditions with fixed periodicity (10-5 m).

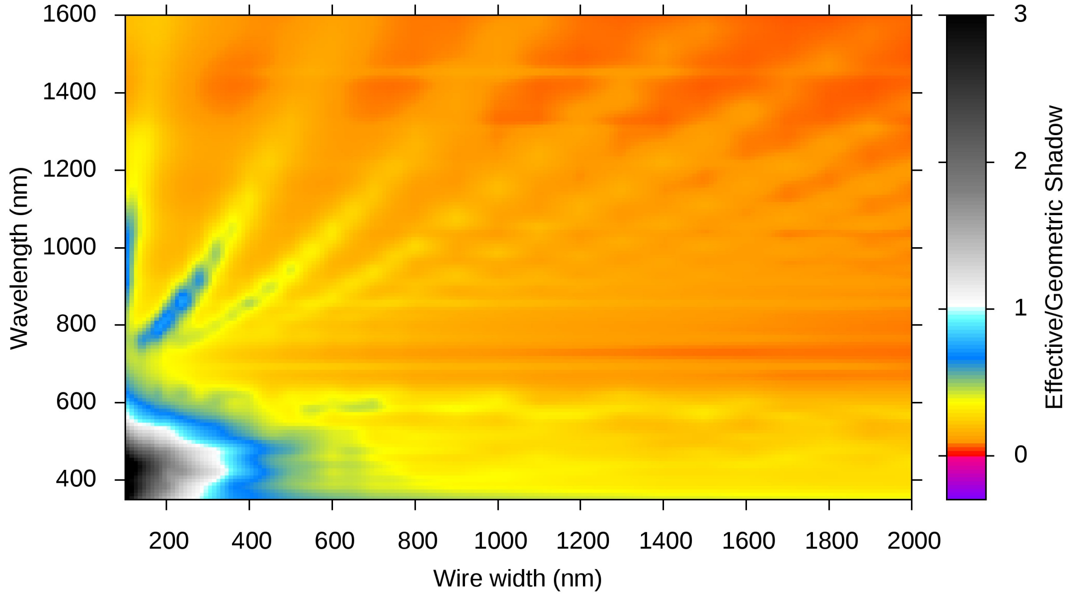

Suppl. Fig. S24. Absorption efficiency for an array of silver wires on a Solar cell. Electric field perpendicular to the wire. Periodic boundary conditions with fixed periodicity (10^{-5} m).

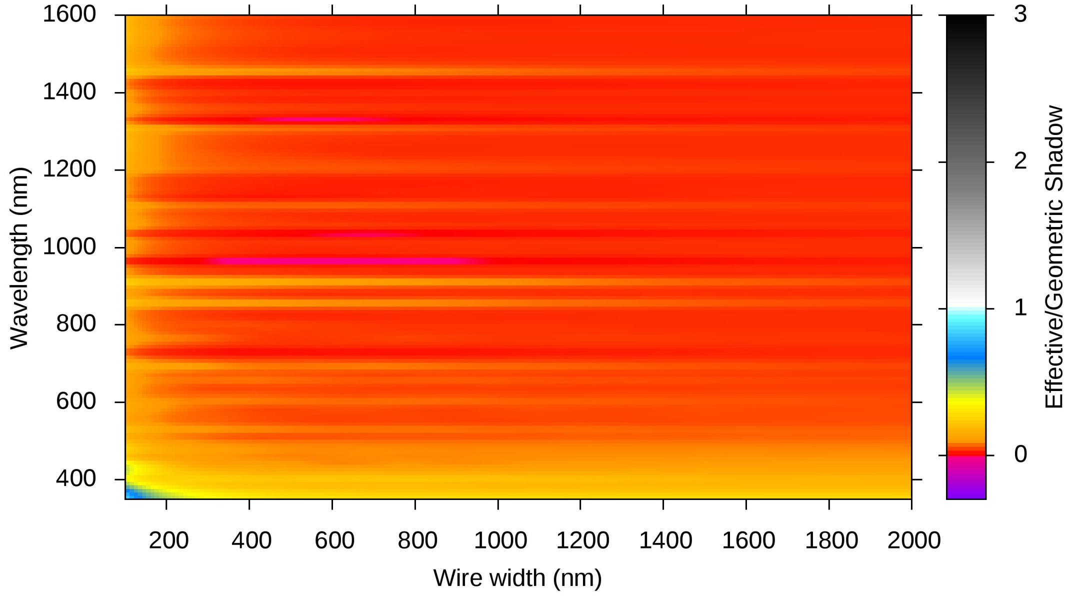

Suppl. Fig. S25. Absorption efficiency for an array of silver wires on a Solar cell. Electric field parallel to the wire. Periodic boundary conditions with fixed periodicity (10^{-5} m).

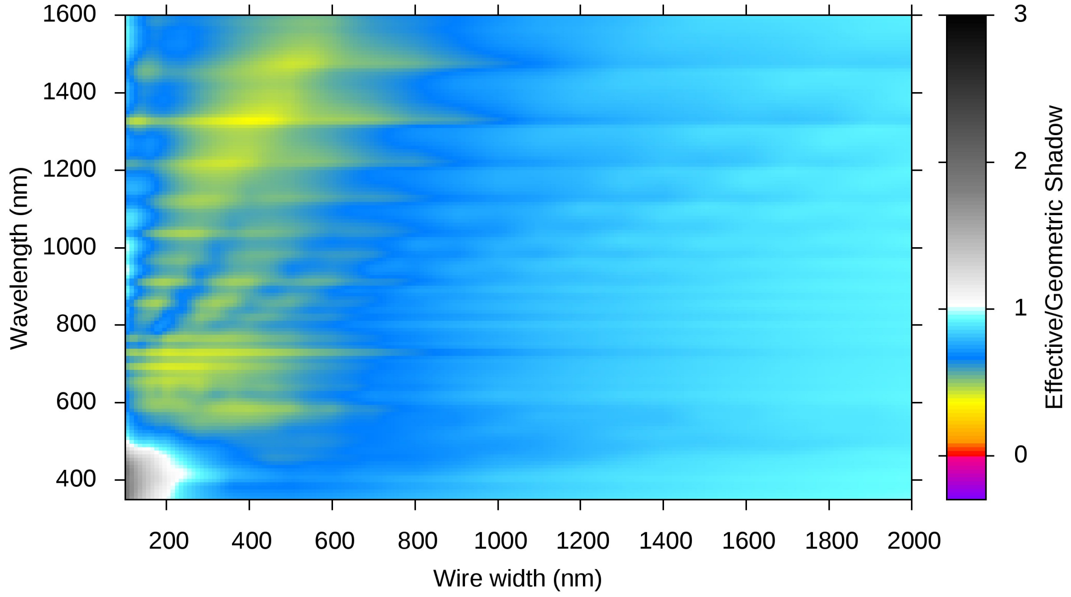

Suppl. Fig. S26. Shadowing efficiency for an array of silver wires on a Solar cell. Unpolarized light. Periodic boundary conditions with fixed periodicity (10^{-5} m).

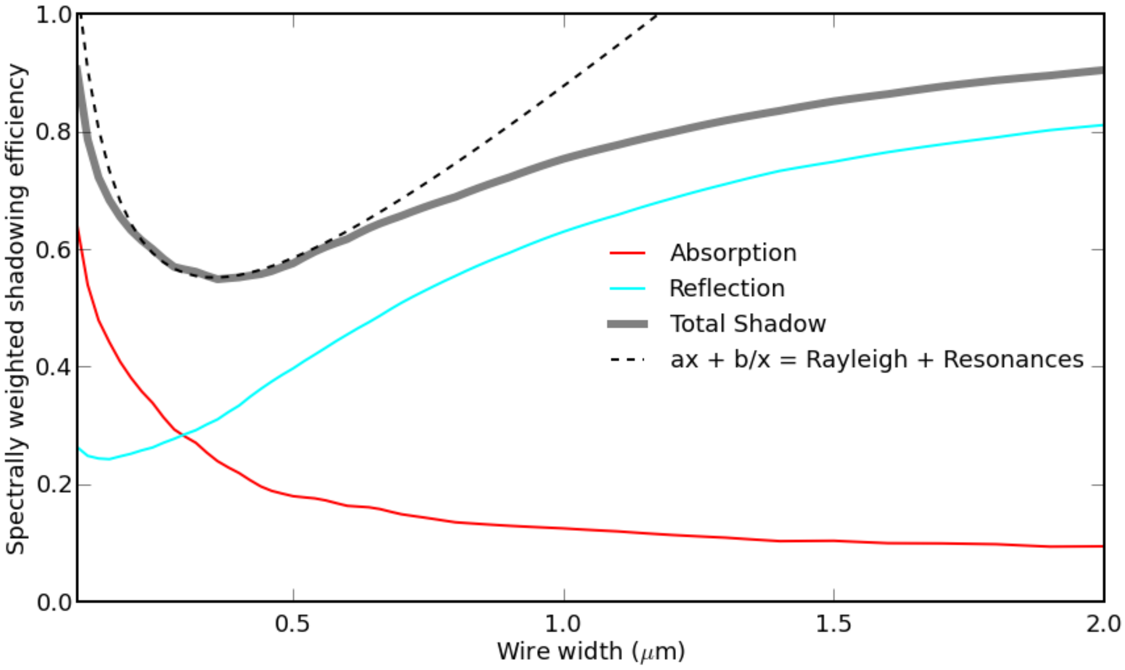

Suppl. Fig. S27. Spectrally weighted shadowing efficiency separated in absorption and reflection contributions for an array of silver wires on a Solar cell. The data are weighted with the direct + circumsolar AM1.5 solar spectrum. Periodic boundary conditions with fixed periodicity (10^{-5} m).

Silver wires on a solar cell. Fixed geometric shadow.

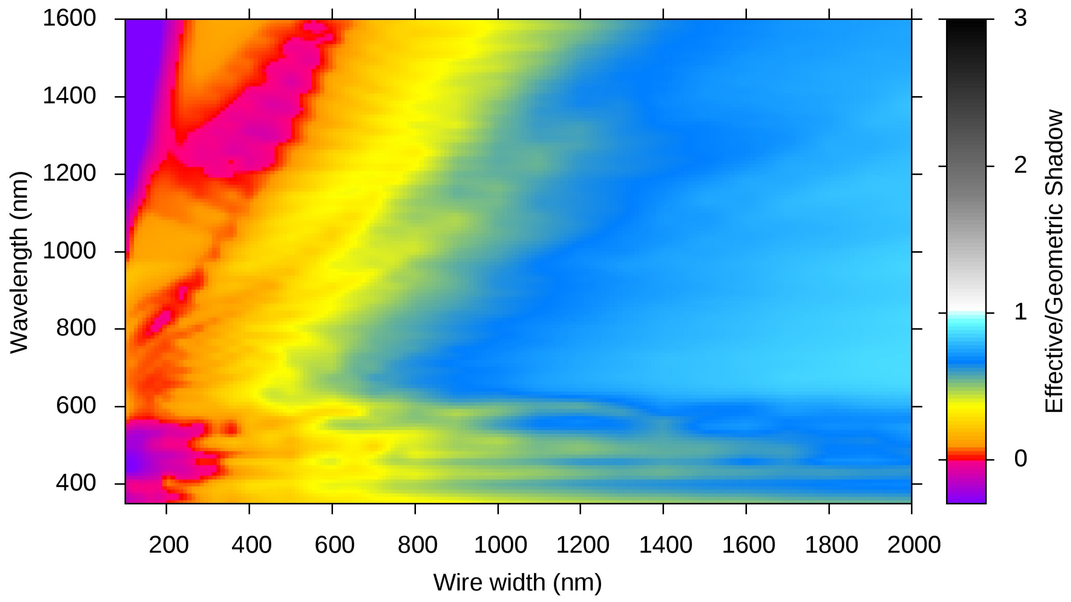

Suppl. Fig. S28. Reflection efficiency for an array of silver wires on a solar cell. Electric field perpendicular to the wire. Periodic boundary conditions with fixed geometric shadow factor (3%).

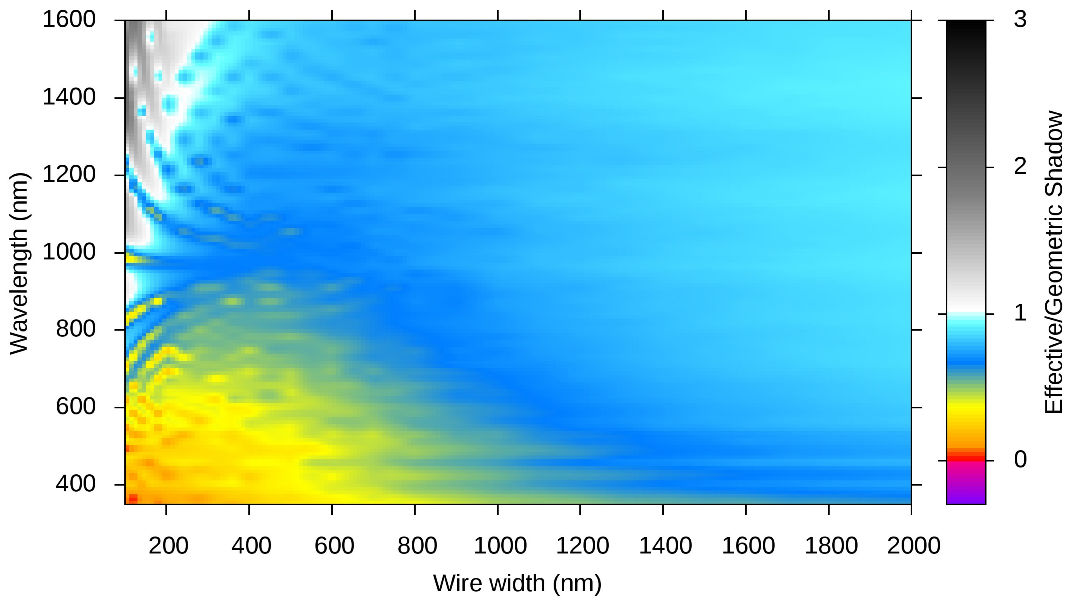

Suppl. Fig. S29. Reflection efficiency for an array of silver wires on a Solar cell. Electric field parallel to the wire. Periodic boundary conditions with geometric shadow factor (3%).

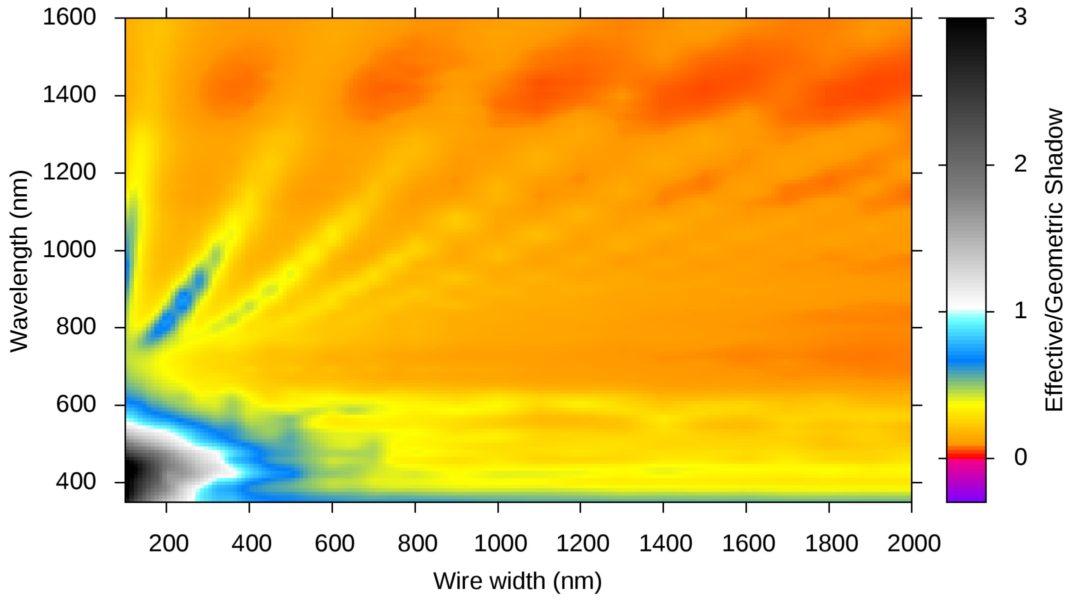

Suppl. Fig. S30. Absorption efficiency for an array of silver wires on a Solar cell. Electric field perpendicular to the wire. Periodic boundary conditions with fixed geometric shadow factor (3%).

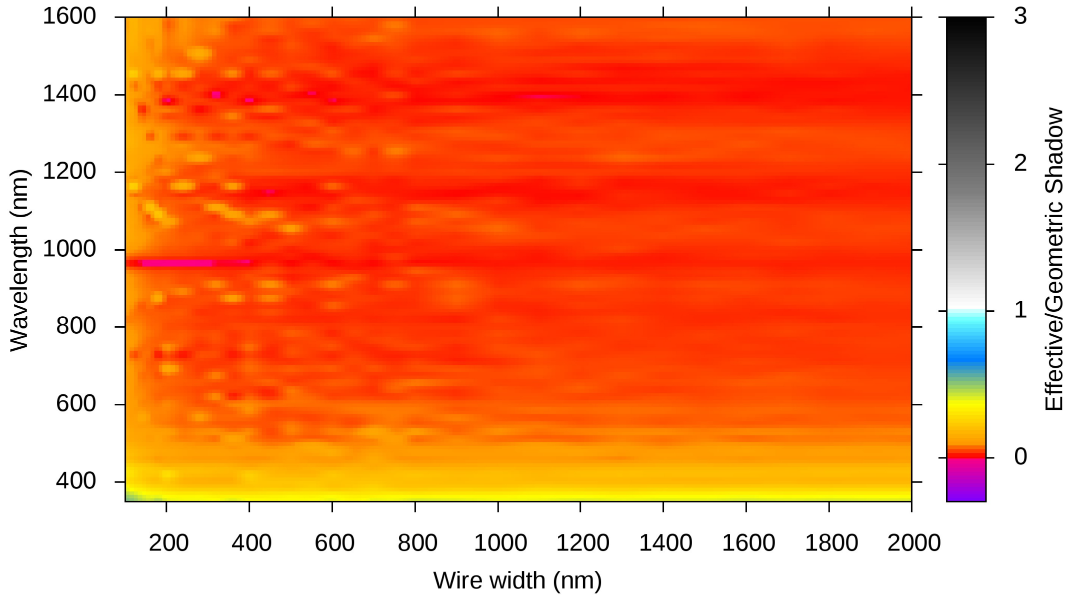

Suppl. Fig. S31. Absorption efficiency for an array of silver wires on a Solar cell. Electric field parallel to the wire. Periodic boundary conditions with fixed geometric shadow factor (3%).

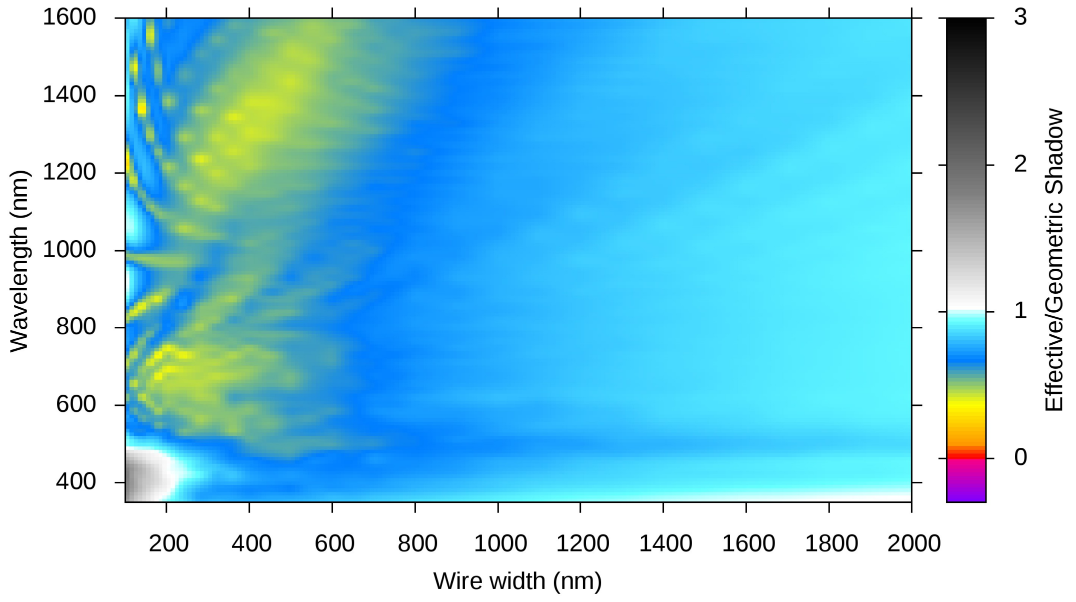

Suppl. Fig. S32. Shadowing efficiency for an array of silver wires on a Solar cell. Unpolarized light. Periodic boundary conditions with fixed geometric shadow factor (3%).

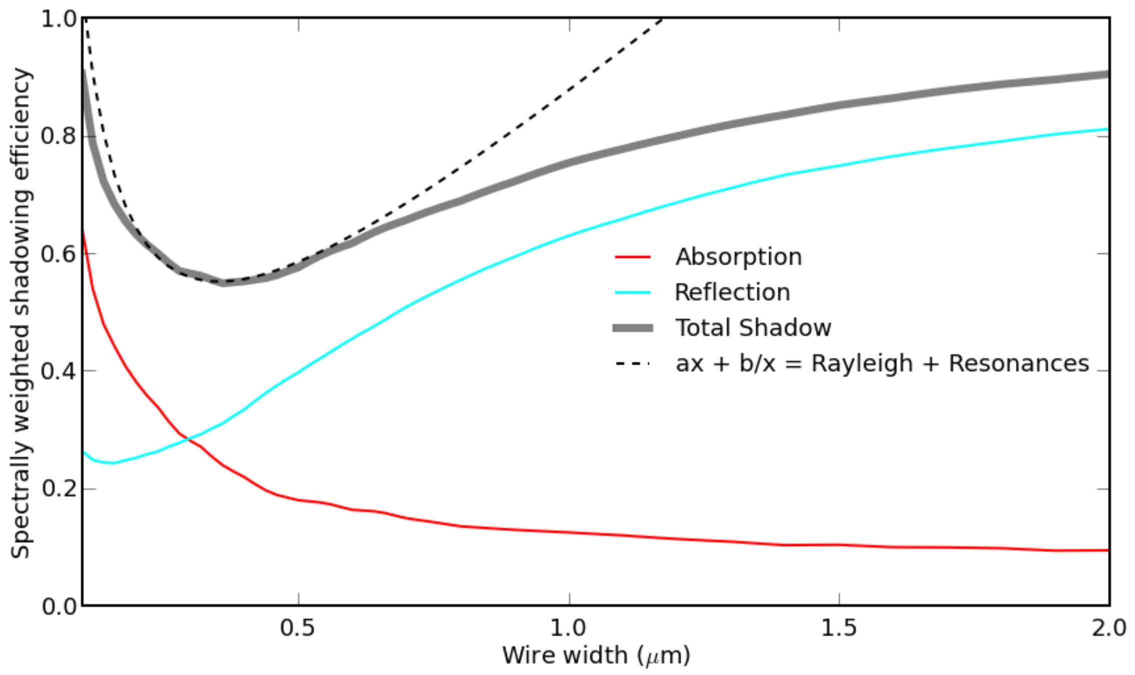

Suppl. Fig. S33. Spectrally weighted shadowing efficiency separated in absorption and reflection contributions for an array of silver wires on a Solar cell. The data are weighted with the direct + circumsolar AM1.5 solar spectrum. Periodic boundary conditions with fixed geometric shadow factor (3%).

Silver wires on a solar cell. Isolated wires

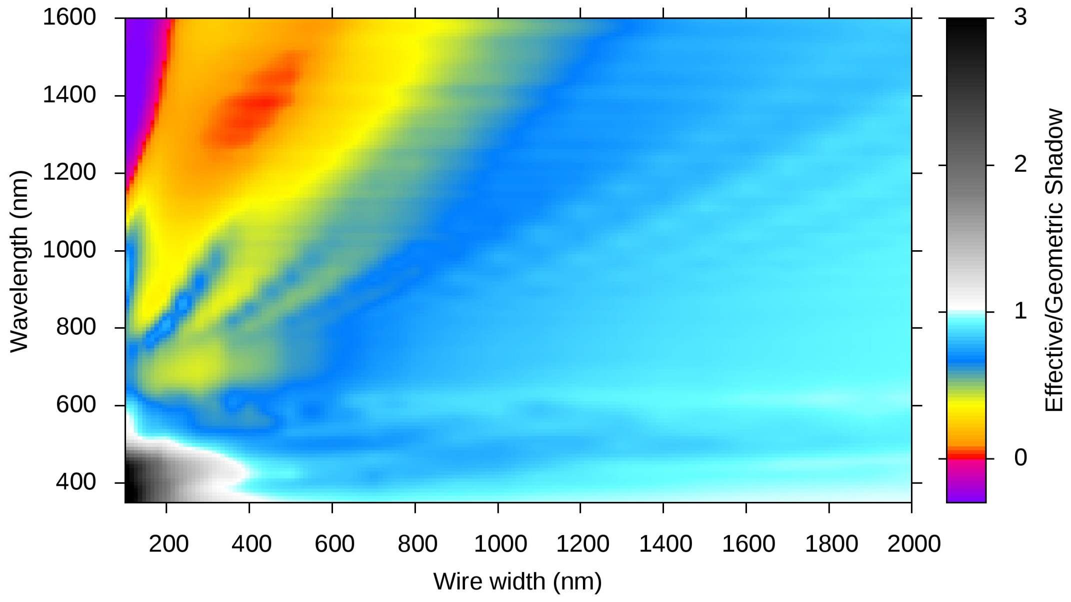

Suppl. Fig. S34. Shadowing efficiency for an isolated silver wire on a solar cell. Electric field perpendicular to the wire.

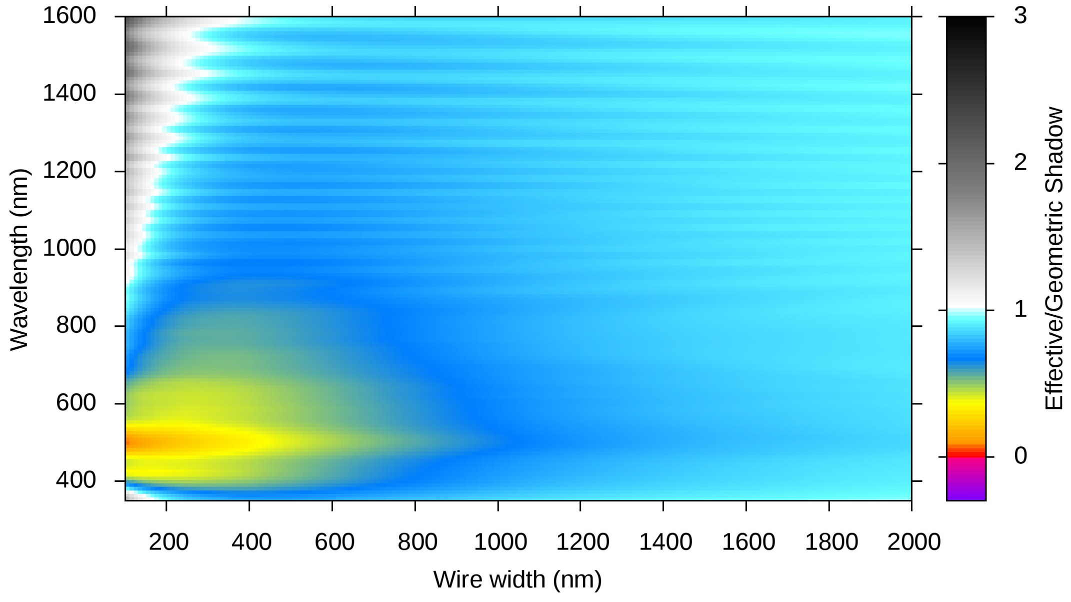

Suppl. Fig. S35. Shadowing efficiency for an isolated silver wire on a solar cell. Electric field parallel to the wire. Periodic boundary conditions with shadow factor (3%).

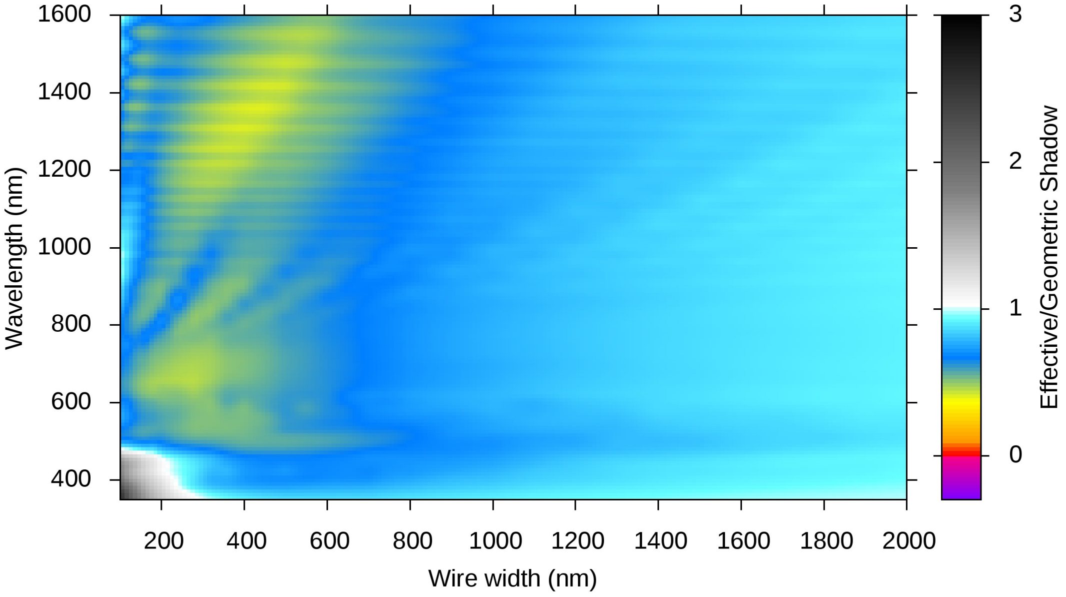

Suppl. Fig. S36. Shadowing efficiency for an isolated silver wire on a solar cell. Unpolarized light.

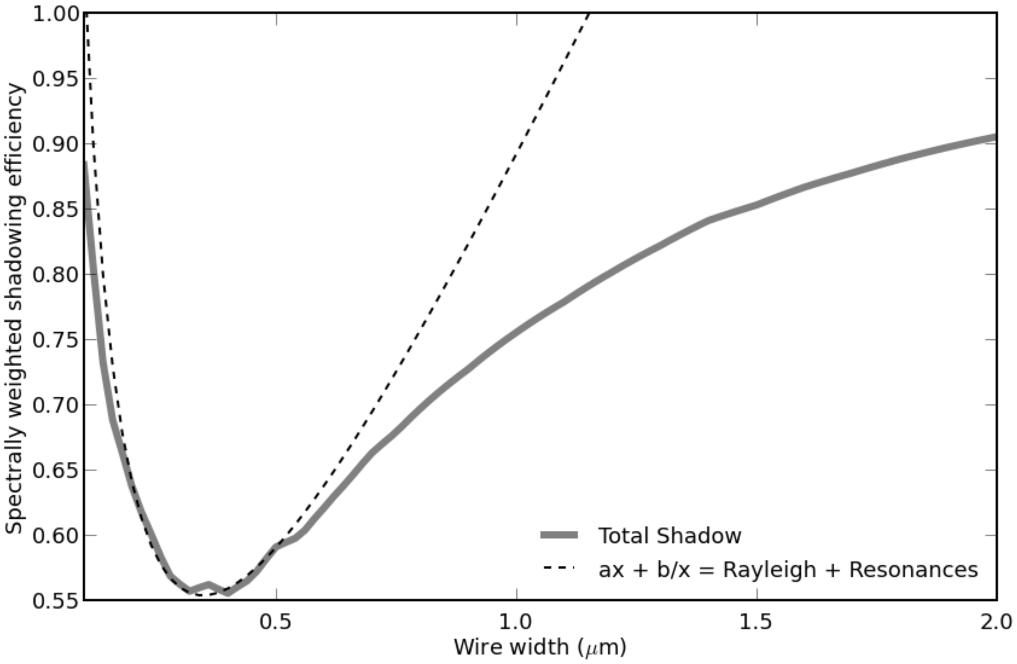

Suppl. Fig. S37. Spectrally weighted shadowing efficiency for an isolated silver wire on a Solar cell. The data are weighted with the direct + circumsolar AM1.5 solar spectrum.

Aluminium wires on a solar cell. Fixed geometric shadow.

Wire height = 100 nm

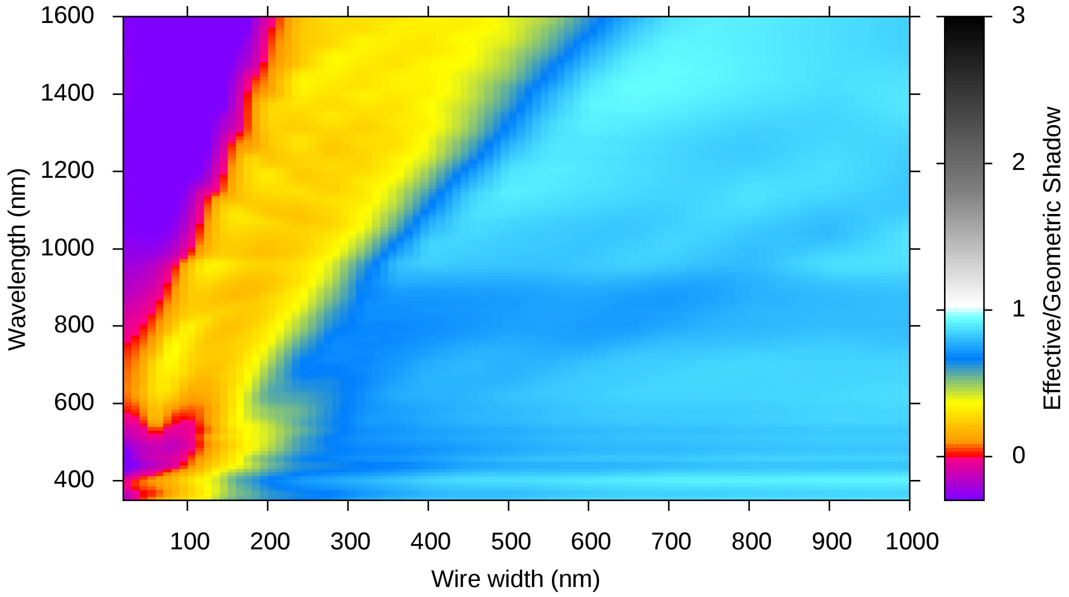

Suppl. Fig. S38. Reflection efficiency for an array of aluminum wires on a solar cell. Electric field perpendicular to the wire. Periodic boundary conditions with fixed geometric shadow factor (3%).

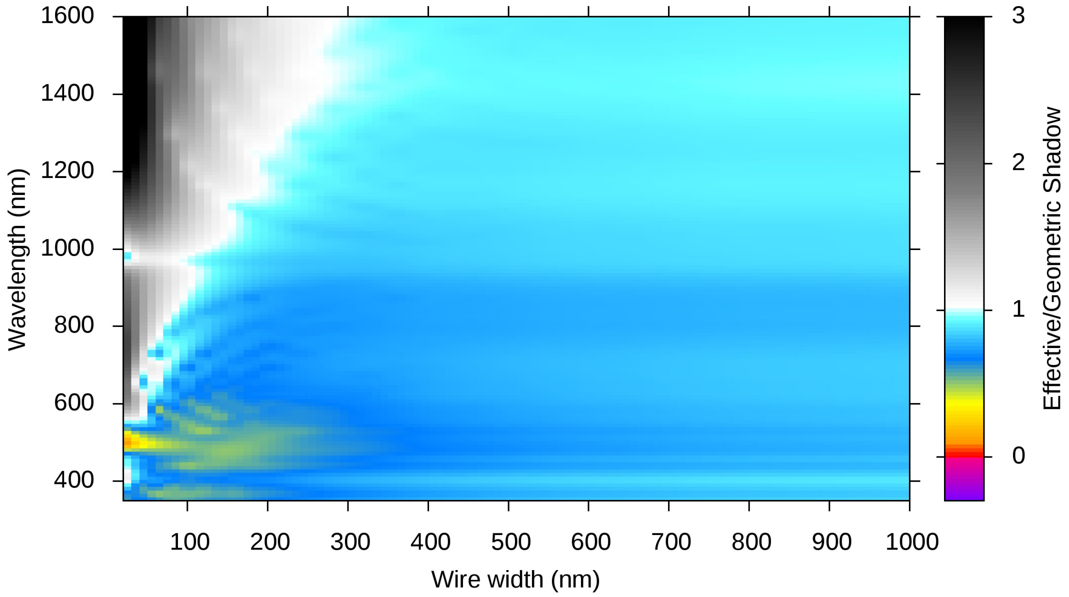

Suppl. Fig. S39. Reflection efficiency for an array of aluminium wires on a Solar cell. Electric field parallel to the wire. Periodic boundary conditions with geometric shadow factor (3%).

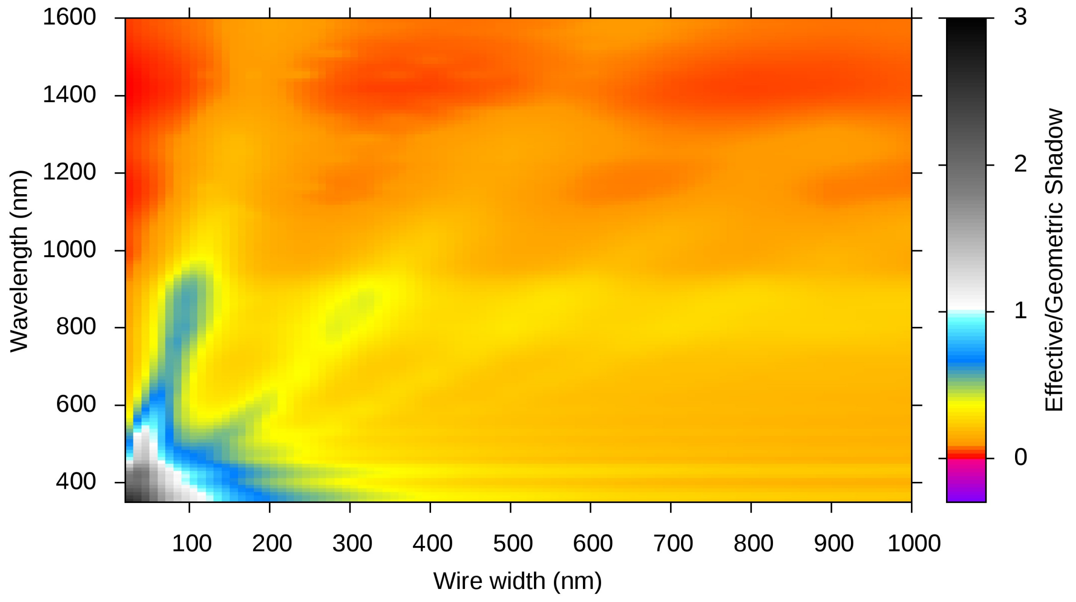

Suppl. Fig. S40. Absorption efficiency for an array of aluminium wires on a Solar cell. Electric field perpendicular to the wire. Periodic boundary conditions with fixed geometric shadow factor (3%).

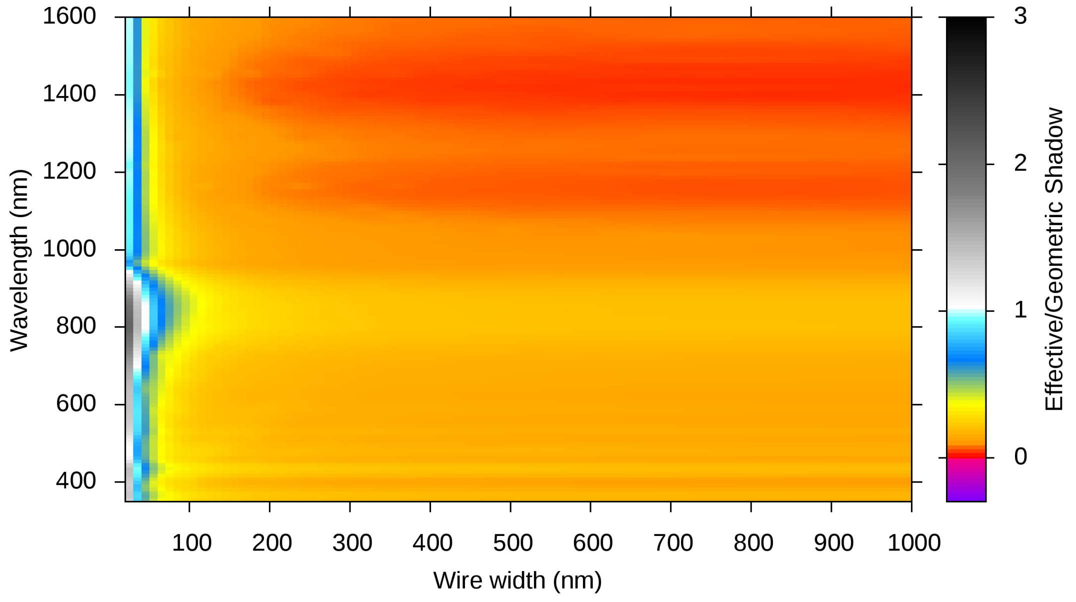

Suppl. Fig. S41. Absorption efficiency for an array of aluminium wires on a Solar cell. Electric field parallel to the wire. Periodic boundary conditions with fixed geometric shadow factor (3%). Note the similarity to Suppl. Fig. S13.

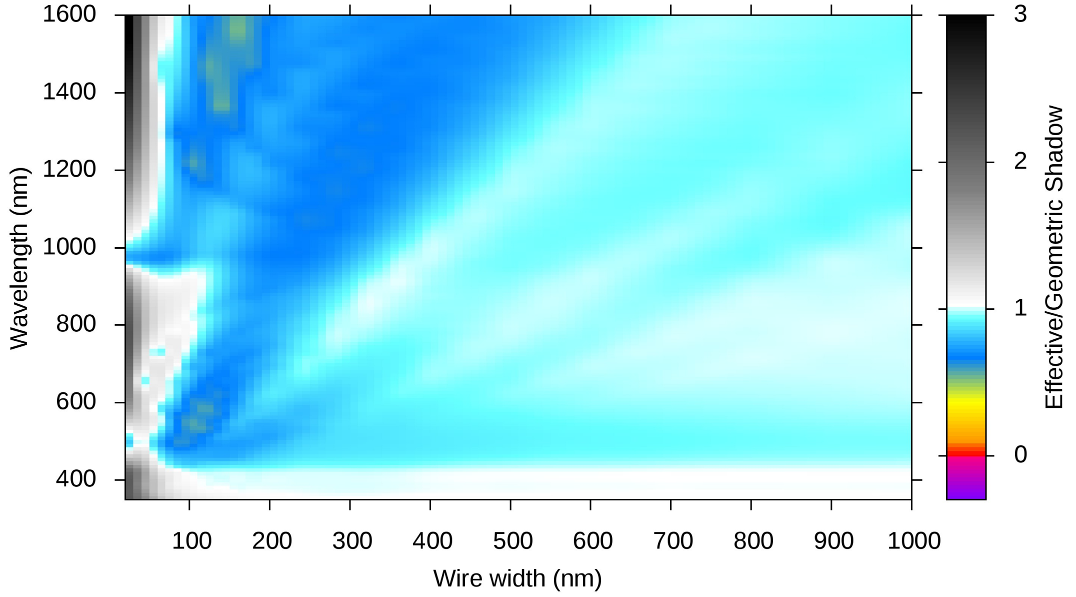

Suppl. Fig. S42. Shadowing efficiency for an array of aluminium wires on a Solar cell. Unpolarized light. Periodic boundary conditions with fixed geometric shadow factor (3%).

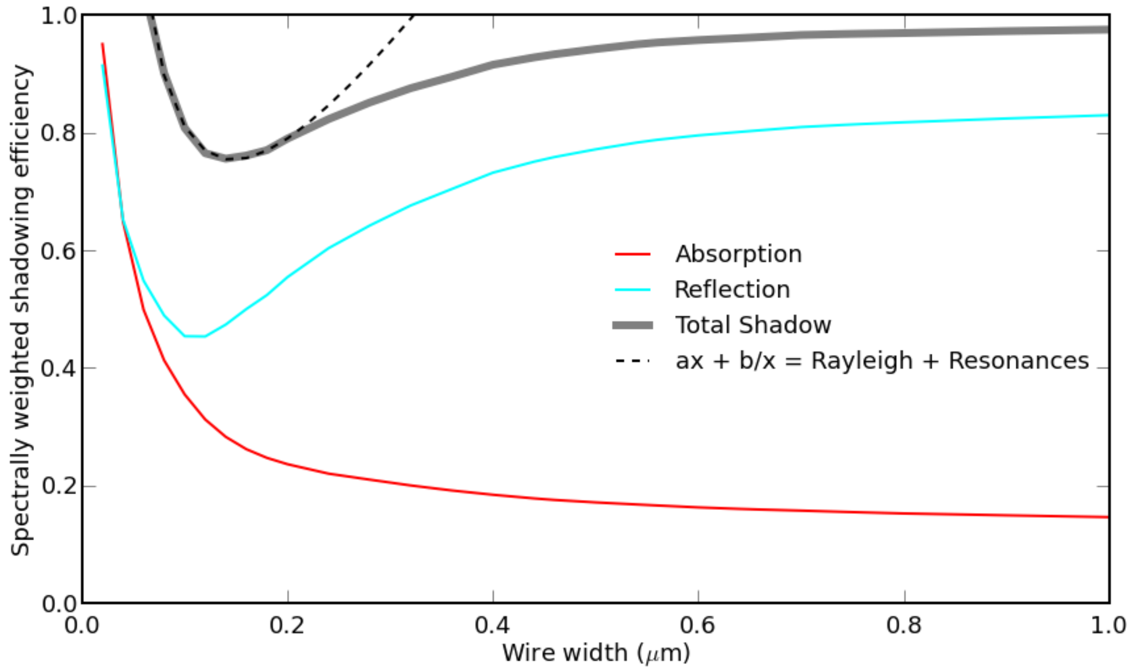

Suppl. Fig. S43. Spectrally weighted shadowing efficiency separated in absorption and reflection contributions for an array of aluminium wires on a Solar cell. The data are weighted with the direct + circumsolar AM1.5 solar spectrum. Periodic boundary conditions with fixed geometric shadow factor (3%).

Video: Steady state near field as a function of wavelength.

The video included as a supplementary material file is the steady state near field as a function of wavelength for transversal polarized light corresponding to the geometry in Supp. Suppl. Fig. S1. Intense parasitic absorption can be observed in the contact layer. In devices without window and contact layers, the near field at the GaAs/Ag interface contributes to the photocurrent, and thus the effect of localized surface plasmon resonances is beneficial rather than detrimental to device energy efficiency.

Device characterization

Quantum efficiency

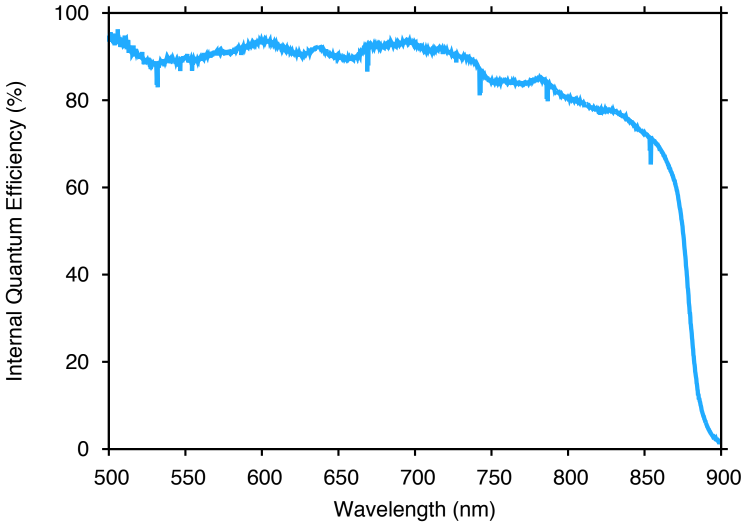

Suppl. Fig. S44. Internal quantum efficiency for our GaAs solar cells. Standard refractive index data has been used to correct for reflection at the SiO2 / GaAs interface.

IV curves

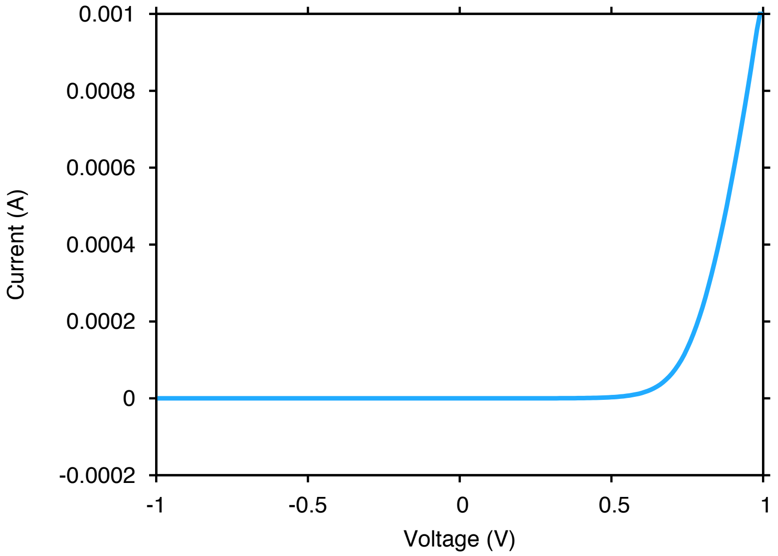

Suppl. Fig. S45. Current voltage characteristic for our GaAs solar cells.
